# SLC40A1 (iron transporter): mechanistic regulation, role in disease pathogenesis, and prospects for targeted therapy

**DOI:** 10.3389/fcell.2026.1816800

**Published:** 2026-05-11

**Authors:** Limian Huang, Lin Pan, Weiling Qin, Yihui Zhao, Yanyun Huang, Xiao Qin

**Affiliations:** 1 Baise People’s Hospital, Baise, Guangxi, China; 2 Affiliated Southwest Hospital of Youjiang Medical University for Nationalities, Baise, Guangxi, China

**Keywords:** disease, ferroptosis, iron homeostasis, iron transporter, SLC40A1

## Abstract

Solute carrier family 40 member 1 (SLC40A1) encodes ferroportin 1 (FPN1), the only known iron efflux transporter in mammalian cells, which is critical for maintaining systemic iron homeostasis and cellular iron balance. SLC40A1 plays a key role in regulating iron homeostasis and is involved in the pathogenesis of inflammatory, fibrotic, neurodegenerative diseases, and cancer, with dysfunction linked to mutations or epigenetic silencing. Its biological functions and regulatory mechanisms are complex and diverse. This article provides an overview of SLC40A1’s molecular and biological properties, elucidates its involvement in disease progression, and evaluates its clinical potential. The study seeks to enhance understanding of SLC40A1’s mechanisms, laying the groundwork for the development of targeted therapies.

## 1 Molecular and biological features of SLC40A1

### 1.1 Gene mapping and analysis of protein structural characteristics

The SLC40A1 gene is located on human chromosome 2 at locus 2q32.2 and encodes ferroportin 1 (FPN1), the primary iron transporter in mammalian cells, commonly referred to by the gene name SLC40A1. FPN1 exhibits the structural characteristics of a transmembrane transporter, containing multiple transmembrane domains, and is recognized as the only known cellular iron export pump in mammals (Hu and Wu, 2021; Duca et al., 2022; Uguen et al., 2023). FPN1 plays a critical role in regulating systemic iron homeostasis (Galy et al., 2024; Fisher et al., 2025). It is prominently expressed in key cell types involved in iron metabolism, including duodenal epithelial cells responsible for dietary iron absorption, hepatocytes for iron storage and release, and macrophages in the reticuloendothelial system, which mediate iron recycling by phagocytosing senescent red blood cells (Billesbølle et al., 2020; Nemeth and Ganz, 2021).

Zyklopen (SLC40A2), a paralog of the canonical iron exporter SLC40A1, is a placenta-enriched transmembrane protein essential for maternal-fetal iron transfer. Distinct from SLC40A1, Zyklopen displays three key characteristics: it lacks sensitivity to hepcidin, thereby mediating constitutive iron efflux independent of systemic iron homeostasis; it harbors a unique C-terminal domain that promotes oxidative stress-induced iron export in neuronal tissues; and it exhibits a highly tissue-specific expression profile, with minimal to undetectable expression in hepatocytes and macrophages (Helman et al., 2021).

### 1.2 Core physiological function: facilitating the efflux of intracellular iron

SLC40A1 (FPN1) serves a well-defined physiological function as an iron transporter, mediating the movement of intracellular ferrous ions (Fe^2+^) from the cytoplasm to the extracellular environment (Feng et al., 2024; Uguen et al., 2024). This iron efflux is not an isolated process; it is functionally coupled with two key multicopper oxidases, indicating a coordinated mechanism (Dlouhy et al., 2019). In duodenal epithelial cells, FPN1 works in tandem with hephaestin (Heph) to export iron, while in other tissues, it partners primarily with soluble ceruloplasmin (sCP), a plasma copper-containing oxidase that aids in iron efflux (Jończy et al., 2020; Yeh et al., 2009). The significance of this coupling is that hephaestin (Heph) and soluble ceruloplasmin (sCP) mediate the extracellular oxidation of ferrous iron (Fe^2+^) exported by FPN1, converting it to ferric iron (Fe^3+^) for binding to transferrin (Tf), the primary plasma iron transport protein (Hu et al., 2023).

**FIGURE 1:**
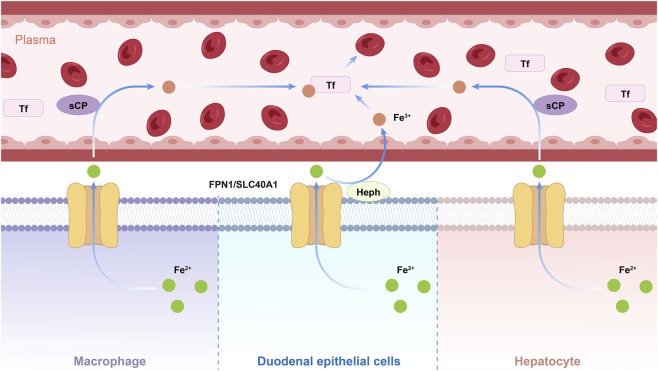
Schematic diagram illustrating Fe^2+^ extrusion from macrophages, duodenal epithelial cells, and hepatocytes mediated by ferroportin 1 (FPN1/SLC40A1).

From a broader physiological perspective, the SLC40A1-mediated iron efflux mechanism facilitates the release of intracellularly stored iron—including iron bound to ferritin in hepatocytes and iron recycled from red blood cells by macrophages—into the bloodstream. This process is essential for meeting the iron demands of key metabolic processes, such as erythropoiesis (red blood cell production) and mitochondrial respiratory chain activity. Impairments in this mechanism can lead to disrupted iron distribution and metabolic dysfunction (Yang et al., 2020) (Figure 1).

### 1.3 Multilayer regulatory mechanism

SLC40A1, a central mediator of cellular iron export, is tightly regulated at multiple levels, including transcriptional, post-transcriptional, epigenetic, and post-translational mechanisms, forming a precise network that adapts to iron metabolic demands across various physiological and pathological conditions.

At the transcriptional level, hepcidin, a liver-produced hormone, acts as the master regulator of SLC40A1 function. In response to iron overload or inflammation, hepcidin expression is upregulated, binding directly to SLC40A1 (FPN) to trigger its internalization, ubiquitination, and lysosomal degradation, thereby inhibiting cellular iron export (Zou et al., 2025). In addition to the classical hepcidin-mediated pathway, other transcription factors regulate SLC40A1 expression. Nrf2, an antioxidant stress-responsive factor, modulates transcription by binding to response elements in the SLC40A1 promoter, linking oxidative stress to iron homeostasis (Ulasov et al., 2022; He et al., 2020). Furthermore, the Ets1 transcription factor family can be activated by specific peptide segments, significantly enhancing SLC40A1 transcriptional activity, positioning it as a potential target for precise regulation of SLC40A1 expression (Abdalla et al., 2025).

Post-transcriptional regulation is primarily mediated by microRNAs (miRNAs), which interact specifically with target mRNAs. Extensive experimental evidence indicates that miR-147a, miR-4735-3p, and miR-18a-5p bind directly to the 3′-untranslated region (3′-UTR) of SLC40A1 mRNA, leading to suppression of SLC40A1 protein expression through translational inhibition or increased mRNA decay (Xu et al., 2022; Zhu et al., 2022). This regulatory mechanism is rapid and plays a critical role in the dysregulation of iron metabolism during acute stress and physiological challenges.

Epigenetic regulation plays a pivotal role in the stable expression of SLC40A1, with promoter region methylation being one of the most extensively studied mechanisms (Ju et al., 2020; Chen et al., 2024). In several cancers, including nasopharyngeal carcinoma (NPC), hypermethylation of the SLC40A1 promoter results in sustained transcriptional silencing. This alteration may contribute to tumor reprogramming by disrupting cellular iron export (Wang et al., 2025). Additionally, histone modifications are crucial in regulating SLC40A1 expression. In inflammatory conditions, histone deacetylases 1 and 3 (HDAC1/3) are recruited to the SLC40A1 promoter, where they remove acetyl groups from histones, inducing chromatin condensation and limiting transcriptional accessibility, thus repressing SLC40A1 expression (Xiao P. et al., 2024). Conversely, specific genetic variants in the SLC40A1 promoter, such as c.-662C > T and c.-8C > G, enhance binding of transcription factors CREB-1 and HLF, leading to increased promoter activity and upregulation of SLC40A1 expression, explaining interindividual differences in SLC40A1 expression (Ha et al., 2024).

At the post-translational level, beyond the hepcidin-mediated ubiquitin-proteasome degradation pathway, emerging evidence highlights selective autophagy as a key regulatory mechanism for modulating SLC40A1 protein levels (Chen et al., 2023; Liu L. et al., 2021). Under ferroptosis-inducing conditions, SQSTM1/p62 binds to SLC40A1, promoting its autophagic-lysosomal degradation. This process enhances intracellular iron accumulation, reinforcing the iron overload–ferroptosis feedback loop (Li J. et al., 2021). Protein interactions and post-translational modifications also directly influence SLC40A1 function. For example, in lung epithelial cells, the β-N-acetylglucosamine (O-GlcNAc)-modified ferritin heavy chain (FTH1) interacts with SLC40A1, inhibiting iron export. This interaction reveals a novel mechanism of cooperation between iron storage and transport proteins (Gu et al., 2025).

**FIGURE 2:**
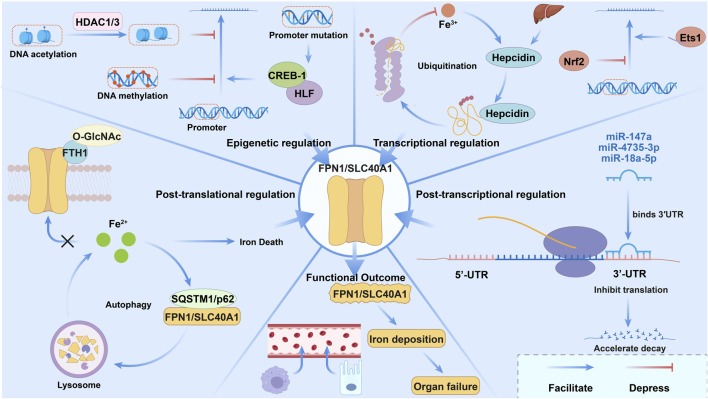
The multi-level regulatory network of SLC40A1/FPN1, encompassing transcriptional, post-transcriptional, epigenetic, and post-translational mechanisms. These pathways coordinately modulate SLC40A1 expression and activity, thereby controlling cellular iron efflux and systemic iron homeostasis. In tumor cells, SLC40A1 exhibits context-dependent, heterogeneous functions: reduced expression in specific cancer types impairs iron efflux and drives iron accumulation, while elevated expression in other malignancies modulates ferroptosis, therapeutic resistance, and tumor progression, as detailed in the manuscript.

In summary, the multi-level regulatory network governing SLC40A1 ensures precise and adaptive systemic iron export through coordinated interactions at various regulatory levels. Disruption of this network can impair iron homeostasis, contributing to the development and progression of pathological conditions such as anemia, iron overload disorders, and cancer. These findings highlight the critical role of this network in disease mechanisms and provide a solid theoretical foundation for the development of targeted therapies aimed at modulating SLC40A1 activity. Refer to Figure 2.

Collectively, the multi-level regulatory network of SLC40A1 enables dynamic, context-dependent control of cellular iron export rather than static expression. Transcriptional, epigenetic, and post-translational mechanisms act coordinately rather than sequentially to adjust SLC40A1 abundance and activity in response to iron status, inflammation, oxidative stress, and genetic background. Dysregulation at any level disrupts iron homeostasis and contributes to anemia, iron overload, and cancer progression. This integrated architecture defines the core biology of SLC40A1 and provides a rational framework for understanding its divergent roles in disease and developing targeted therapeutic interventions.

## 2 SLC40A1 expression and function in disease

The functional roles of SLC40A1 (FPN1) exhibit significant variability across different disease contexts, with contrasting roles observed in non-tumor versus tumor diseases. These differences are critically linked to its central function in regulating cellular iron homeostasis (Table 1).

**TABLE 1:** Summary of SLC40A1 expression patterns, upstream regulatory mechanisms, and core iron-mediated pathogenic cascades across representative non-tumor and tumor diseases.

Classification	Disease	Pathway	Mechanism	Clinical sample validation	Sample size	Cell line/animal model	References
Neoplastic	Glioblastoma	miR-147a-SLC40A1	miR-147a activation → SLC40A1 inhibition → ferroptosis activation → TMZ sensitization	Yes	180	Cell line and animal model	Xu et al. (2022)
	Clear cell carcinoma of kidney	miR-4735-3p-SLC40A1	miR-4735-3p activation → SLC40A1 inhibition → ferroptosis activation → migration inhibition	Yes	50	Cell line	Zhu et al. (2022)
	Prostatic cancer	Leo-SLC40A1	Alkaloid from Leonurus japonicus activation→SLC40A1 activation → activates ferroptosis → inhibits transplanted tumor	Yes	30	Cell line and animal model	Liang et al. (2022)
	Pancreatic cancer	Na-OHB-CAV1-SLC40A1	Ketone metabolites inhibit CAV1 → SLC40A1 inhibition → ferroptosis activation → survival extension	Yes	24	KPC mice	Liang et al. (2025)
	Lung cancer	Sarcosine-MXD3-SLC40A1	Sarcosine inhibits SLC40A1 → Inhibits iron efflux → Activates ferroptosis → Sensitizes to cisplatin	Yes	52	Cell line	Shan et al. (2025)
	Lung cancer	Pomolic acid - SLC40A1	Pomolic acid inhibits SLC40A1 → GSH inhibition → ferroptosis activation	Yes	60	Cell line and animal model	Ji et al. (2025)
	Esophageal squamous carcinoma	Bru-NRF2-SLC40A1	Brucea javanica oil inhibits NRF2→ SLC40A1 suppression→ activates ferroptosis→ inhibits tumor growth	Yes	30	Cell line and animal model	Zhu et al. (2023)
	Breast cancer	β-eudesmol-MEK/ERK-SLC40A1	β-eudesmol inhibits SLC40A1 → ferroptosis activation → proliferation inhibition	Yes	<50	cell line	Li Z. et al. (2024)
	Leukemia	Cobalt-based nanomaterials-HMOX1-SLC40A1	Nanomaterials inhibit SLC40A1 → Fe^2+^ activation → ferroptosis activation → radiosensitization	Yes	30	Cell line and animal model	Zhao et al. (2023)
	Nasopharynx cancer	IGF2BP2-m6A-CP-SLC40A1	IGF2BP2 inhibits CP → SLC40A1 is inhibited → ferroptosis is blocked → promoting cancer	Yes	<50	Cell line and animal model	Yuan et al. (2025)
Non-neoplastic	Diabetic Nephropathy	Acupuncture - SLC40A1	Acupuncture activates SLC40A1 → inhibits podocyte ferroptosis → inhibits proteinuria	Yes	30	Diabetic kidney disease rat model	Yue et al. (2024)
​	Alzheimer disease	Jbs-5YP-Ets1-Slc40a1	Peptide phosphorylation Ets1 → Slc40a1 activation → brain iron inhibition → cognitive improvement	Yes	20	Alzheimer’s disease mouse model	Zou et al. (2025)
	Anemia of inflammation	Butyric acid - HDAC - Slc40a1	Butyric acid inhibits HDAC → Macrophage Slc40a1 activation → Iron release activation → Anemia relief	Yes	>50	Cell line and animal model	Xiao P. et al. (2024)
	ALI/ARDS	Sivelestat-SLC40A1	Sivelestat activates SLC40A1 → inhibits endothelial ferroptosis → reduces lung injury	Yes	30	Mouse LPS-induced acute lung injury model	Ren et al. (2024)
	Aortic dissection	GJA1/SNCA-SLC40A1	SLC40A1 inhibition → activation of ferroptosis in VSMCs → progression of dissection	Yes	30	Cell line and animal model	Zhao et al. (2024)
	Spinal cord injury	Fer-1-ERK/SP1-SLC40A1	Fer-1 activates SLC40A1 → inhibits neuronal ferroptosis → restores motor function	Yes	30	Rat SCIRI model	Li S. et al. (2024)
	ICH	Cinnamaldehyde-SLC40A1	Cinnamaldehyde activates SLC40A1 → iron efflux is activated → ferroptosis is inhibited → neuroprotection is achieved	Yes	30	Mouse ICH model	Liu Y. et al. (2025)
	OSA	DEIRG-SLC40A1	SLC40A1 inhibition → abnormal memory B cells → immune imbalance in OSA	Yes	206 cases vs. 420 controls	PBLs	Zhou et al. (2025)
	SSNHL	SLC40A1-8CG variant	SLC40A1 variation → imbalance of iron homeostasis → activation of hearing loss risk	Yes	206 cases vs. 420 controls	PBLs	Tisato et al. (2023)
	Hereditary hemochromatosis Type 4B	SLC40A1 p.Tyr333His	Gain-of-function mutations → iron overload → effective treatment with red blood cell apheresis and deferasirox	Yes	2 (father and daughter)	PBLs with functional validation	Hu et al. (2023)

### 2.1 Pathogenic role in non-neoplastic diseases

In non-malignant diseases, impaired function or reduced activity of SLC40A1 is the central pathogenic mechanism, directly leading to intracellular iron retention. This iron accumulation promotes the generation of iron-dependent reactive oxygen species (ROS), inducing oxidative stress, cellular damage, and accelerating disease progression.

#### 2.1.1 Hereditary hemochromatosis (HH) and rare hematological disorders

Hereditary hemochromatosis type 4, an autosomal dominant genetic disorder (Kane et al., 2021; Britton and Bacon, 2002; Babitt and Lin, 2011), is caused by pathogenic mutations in the SLC40A1 gene.SLC40A1 encodes ferroportin, the primary cellular iron exporter, which coordinates systemic iron homeostasis by mediating ferrous iron efflux from cells. These variants are the exclusive cause of ferroportin disease, also known as hereditary hemochromatosis type 4 (HH type 4), one of the rarer subtypes of hereditary iron overload disorders (Li Y. et al., 2024; Zhang et al., 2019). Based on the impact of these mutations on SLC40A1 (FPN1) protein activity and associated clinical phenotypes, they are categorized into two primary types.

Loss-of-function mutations in SLC40A1, associated with Hereditary Hemochromatosis type 4A, include recurrent hotspot variants such as p.Tyr333His and p.Thr419Ile (p.T419I). The Tyr333His mutation disrupts the iron-coordinating residues within the transmembrane domain, while the Thr419Ile variant alters the conformation of the extracellular loop, indirectly impairing iron export efficiency (Callebaut et al., 2014). The defective SLC40A1 protein fails to effectively export intracellular iron, leading to substantial iron accumulation in macrophages, particularly reticuloendothelial macrophages in the spleen and liver, as well as persistently elevated serum ferritin levels. This clinical biochemical profile is characteristic of HH type 4A (Kawabata, 2018).

Gain-of-function/hepcidin resistance mutations (HH type 4B), such as p.Cys326Ser, preserve iron transport activity in SLC40A1 but induce structural changes in the hepcidin-binding domain, resulting in resistance to hepcidin-induced ubiquitination and proteasomal degradation (Honma et al., 2021). This resistance effect maintains the expression of SLC40A1 on the surface of duodenal epithelial cells and macrophages, leading to unchecked iron efflux into the systemic circulation. As a result, serum Tf saturation increases significantly, causing progressive iron deposition in vital organs such as the liver and heart.

The genetic landscape of SLC40A1 mutations is highly heterogeneous. Over 140 distinct genetic variants have been identified, each linked to varying degrees of clinical severity. For example, the splice site mutation c.1402G > A in SLC40A1 causes exon skipping during transcription, but the resulting truncated protein retains partial iron transport activity. This residual function explains the mild to moderate iron overload phenotypes seen in affected individuals, in contrast to the severe clinical manifestations typically associated with classic loss-of-function mutations (Wu et al., 2022).

SLC40A1 mutations are also increasingly associated with hematological malignancies. A novel p.T419I mutation was identified in a patient with large granular lymphocytic leukemia (LGL leukemia) and pure red cell aplasia, overlapping with the hotspot mutation found in HH type 4A.

This study suggests that intracellular iron accumulation due to SLC40A1 mutations may promote the clonal expansion of leukemia cells by activating key pro-proliferative intracellular signaling pathways, such as the MAPK/ERK pathway. These findings propose that SLC40A1 dysfunction could serve as a critical molecular link in the progression from benign hematological disorders to malignant hematopoietic diseases (Tertre et al., 2021).

#### 2.1.2 SLC40A1 is dysregulated in inflammatory and iron-overload anemia

The inflammatory response is a key factor in the suppression of SLC40A1 function (Wei et al., 2022). The core mechanism involves the sequestration of iron within macrophages, limiting the availability of circulating iron and playing a critical role in the development of anemia of chronic disease (ACD) (Marques et al., 2025; Liu S. et al., 2025).

In inflammatory bowel disease (IBD), metabolic disturbances in the intestinal microbiota lead to a significant reduction in fecal butyrate levels (Li G. et al., 2021). Butyrate deficiency promotes the recruitment of HDAC1/3 to the SLC40A1 gene promoter, resulting in transcriptional repression and the downregulation of ferroportin expression. This impairment disrupts iron efflux from intestinal macrophages, exacerbating iron deficiency anemia. Conversely, studies on exogenous intervention have shown that alleviating HDAC1/3-mediated transcriptional repression of SLC40A1 and restoring its iron transport function not only ameliorates anemia in IBD but also significantly attenuates intestinal inflammation by suppressing the expression of the pro-inflammatory cytokine TNF-α (Xiao P. et al., 2024; Peng et al., 2024).

Similarly, various pro-inflammatory stimuli, such as TLR2/4 ligands, lipoteichoic acid (LTA), and heat-inactivated *Staphylococcus aureus*, rapidly downregulate SLC40A1 mRNA expression via activation of the NF-κB–HDAC signaling axis, impairing cellular iron export. In contrast, pan-HDAC inhibitors counteract this downregulation by inhibiting HDAC activity, restoring iron export function and highlighting their potential as therapeutic interventions for inflammatory anemia (Marques et al., 2025).

#### 2.1.3 SLC40A1 downregulation in acute tissue injury and its role in ferroptosis regulation

Downregulation of SLC40A1 expression is a widespread pathological feature across diverse models of acute organ injury (Hao et al., 2021). Functional impairment of SLC40A1 promotes intracellular iron accumulation, activating the ferroptosis pathway and contributing to cellular and tissue damage. This mechanistic link is evident in conditions such as sepsis and spinal cord injury.

Sepsis-associated myocardial injury: In an LPS-induced rat model of sepsis-related cardiomyopathy, SLC40A1 (FPN) expression was markedly downregulated in cardiomyocytes, leading to significant intracellular Fe^2+^ accumulation and depletion of glutathione peroxidase 4 (GPX4), a key enzyme that prevents ferroptosis. Further research demonstrated that the ferroptosis inhibitor ferrostatin-1 (Fer-1) effectively suppresses ferroptosis in cardiomyocytes by modulating the TLR4/NF-κB signaling pathway, significantly improving left ventricular ejection fraction and alleviating key manifestations of cardiac dysfunction (Xiao et al., 2021).

Spinal cord ischemia-reperfusion injury (SCIRI): In the SCIRI model, neuronal ferroptosis plays a central role in spinal cord neurological dysfunction. Fer-1 suppresses neuronal ferroptosis by upregulating the SLC40A1-GPX4 signaling axis, with activation of the ERK1/2-SP1 pathway enhancing GPX4 gene transcription and restoring the iron export function mediated by SLC40A1, leading to a dual inhibitory effect on ferroptosis (Li S. et al., 2024).

Other acute injuries: In experimental models of cerebral hemorrhage and retinal degeneration, neuroprotective effects are closely linked to SLC40A1. For example, cinnamaldehyde alleviates iron accumulation by restoring SLC40A1 expression in intracerebral hemorrhage models, while melatonin mitigates visual impairment associated with retinal degeneration by suppressing lipid peroxidation—a hallmark of ferroptosis—in retinal cells and by synergistically modulating SLC40A1 function (Liu Y. et al., 2025; Wei et al., 2022).

#### 2.1.4 The protective role of SLC40A1 in metabolic syndrome and organ fibrosis

In the context of metabolic diseases, SLC40A1 plays a pivotal protective role in mitigating end-organ damage, such as renal injury and hepatic fibrosis, by maintaining cellular iron homeostasis. Its functional impairment is a significant contributing factor to organ damage associated with metabolic syndrome (Zhang et al., 2024).

Diabetic kidney disease (DKD): Podocyte ferroptosis is a key mechanism in the development of proteinuria during DKD progression. Acupuncture treatment or administration of selenium-containing nanoparticles can effectively mitigate proteinuria and delay renal injury progression in preclinical DKD models. These effects are achieved through the upregulation of SLC40A1 expression in renal tissues, inhibition of the TfRc/NCOA4-mediated iron phagocytosis pathway, and reduction of iron overload and ferroptosis in podocytes (Yue et al., 2024).

Hepatic fibrosis: Transcriptional regulation of SLC40A1 represents a critical therapeutic target for hepatic fibrosis intervention. Nrf2 activators, such as Bitopertin, enhance SLC40A1 expression through activation of the Nrf2 signaling pathway. This promotes iron export from hepatocytes and hepatic stellate cells, reduces intracellular iron accumulation, suppresses hepatic stellate cell activation, reduces collagen deposition, and attenuates liver fibrosis progression. Additionally, intestinal probiotics, such as Akkermansia muciniphila, and their secreted supernatants have been shown to attenuate CCl_4_-induced liver fibrosis in murine models by activating the Nrf2-SLC40A1 signaling pathway (Xiao Z. et al., 2024).

#### 2.1.5 Dysfunction of SLC40A1 in neurodegenerative and sensory disorders

Disordered iron export from the brain is a key pathological hallmark of neurodegenerative diseases, including Alzheimer’s disease (AD) (Wang et al., 2022; Raha et al., 2022). SLC40A1, the primary iron export protein in the central nervous system, is critical for neuronal iron homeostasis, and its dysfunction contributes directly to neurodegeneration (Bao et al., 2021). Moreover, genetic variations or altered expression of SLC40A1 have been strongly linked to sensory disorders.

AD: In aged mouse models of AD, SLC40A1 transcript levels in the hippocampus were significantly downregulated, leading to iron accumulation in hippocampal neurons. This iron overload promotes the generation of iron-dependent free radicals, exacerbating neuronal oxidative stress and synaptic dysfunction. Recent studies have shown that the Jbs-5YP peptide restores SLC40A1 expression in the hippocampus via phosphorylation of the transcription factor Ets1, which reduces iron accumulation and improves cognitive function in AD mouse models (Zou et al., 2025).

Sudden sensorineural hearing loss (SSNHL): A case-control study involving 626 participants revealed that the SLC40A1-8CG homozygous genotype was significantly more prevalent in individuals with SSNHL (8.25%) compared to healthy controls (2.6%), with an odds ratio of 3.34, indicating an elevated risk for the disease. Mechanistic evidence suggests that this genotype may interact with LINE-1 hypomethylation, exacerbating dysregulated iron metabolism in inner ear tissues and contributing to increased severity of hearing impairment (Tisato et al., 2023).

Friedreich’s ataxia (FA): Evidence from patient-derived blood samples and mouse models of FA shows that SLC40A1 expression is inversely correlated with disease severity, with lower expression observed in more advanced stages. These findings suggest that monitoring SLC40A1 expression in peripheral blood could serve as a non-invasive biomarker for tracking disease progression, supporting clinical decision-making and therapeutic monitoring (Koka et al., 2024).

#### 2.1.6 Dysfunction of SLC40A1 in pulmonary diseases

SLC40A1 dysfunction is closely linked to the pathogenesis and progression of chronic lung diseases, including chronic obstructive pulmonary disease (COPD), and acute lung injuries (ALIs) such as acute respiratory distress syndrome (ARDS). The regulatory mechanisms behind this dysfunction involve multiple factors, including cigarette smoke exposure, inflammatory responses, and disruption of iron homeostasis.

Emphysema, a major pathological manifestation of COPD (Cerveri and Brusasco, 2010; Guo et al., 2022), remains incompletely understood (Xu et al., 2023; Chesseron et al., 2023). Tobacco smoking is the primary cause of COPD (Celli et al., 2025; Liu Z. et al., 2025; Upadhyay et al., 2023), and cigarette smoke exposure significantly reduces SLC40A1 (FPN) expression in alveolar epithelial cells (Ju et al., 2025). Additionally, FTH1 modulates O-linked N-acetylglucosamine (O-GlcNAc) post-translational modification, leading to suppressed SLC40A1 translation and impaired iron export in alveolar epithelial cells. In contrast, overexpression of FTH1 via AAV5 vector administration reverses this effect, restores alveolar iron homeostasis, and improves emphysema-like pathological changes (Gu et al., 2025).

In ALI and ARDS, the LPS-induced ALI model demonstrates that sivelestat upregulates SLC40A1 expression in lung tissue 8 h post-inflammation, promoting iron export from pulmonary microvascular endothelial cells, reducing iron-dependent oxidative stress, and alleviating microvascular endothelial barrier dysfunction. Notably, HH mouse models with iron overload show a diminished pulmonary inflammatory response after LPS exposure. These findings suggest a potential biphasic “iron-immune” regulatory relationship between iron metabolism and immune-inflammatory responses, although the underlying mechanisms require further investigation (Ren et al., 2024).

In obstructive sleep apnea (OSA), a common disorder characterized by disrupted breathing and multi-organ dysfunction (McNicholas and Pevernagie, 2022; Lv et al., 2023; Horner, 2023), transcriptome analysis and bioinformatics screening identified SLC40A1 as one of 11 core immune-related genes associated with OSA. Its expression correlates significantly with the infiltration of memory B cells and resting mast cells in the airway tissues of patients with OSA. SLC40A1 may serve as a biomarker in sputum or blood for assessing OSA severity, offering a new target for diagnosis and prognosis (Zhou et al., 2025).

### 2.2 Neoplastic disease

Expression patterns and regulatory mechanisms of SLC40A1 across various tumor types.

#### 2.2.1 Glioblastoma

Glioblastoma (GBM), one of the most prevalent malignant brain tumors, continues to have a poor prognosis for patients (Brown et al., 2022; Sipos et al., 2025; van Dijck et al., 2025). SLC40A1 expression in GBM is closely linked to patient outcomes and is influenced by molecular regulation and cellular changes in the tumor microenvironment, highlighting its role in iron metabolism dysregulation and GBM progression. Recent comprehensive reviews further highlight the centrality of iron metabolism and ferroptosis in glioblastoma progression and therapeutic resistance. As underscored by Caverzan et al. (Caverzan and Ibarra, 2024), glioblastoma cells exhibit profound iron addiction, driven by aberrantly high expression of transferrin receptor 1 (TfR1) to sustain rapid proliferation, DNA synthesis, and angiogenesis. This dysregulated iron uptake not only fuels tumor growth but also renders glioblastoma uniquely vulnerable to ferroptosis—an iron-dependent form of regulated cell death characterized by lethal lipid peroxidation. Emerging therapeutic strategies capitalize on this vulnerability, encompassing iron chelation, direct induction of ferroptosis via inhibition of antioxidant systems (e.g., GPX4), and advanced nanoparticle-based delivery platforms designed to overcome the blood-brain barrier and enhance target specificity. Importantly, these iron-targeted approaches hold significant promise for circumventing conventional therapy resistance and improving outcomes when integrated with standard-of-care treatments.

From a clinical perspective, SLC40A1 expression has emerged as a significant prognostic biomarker for GBM. Accumulating clinical evidence consistently shows that higher SLC40A1 expression in GBM tumor tissues is strongly associated with prolonged overall survival (OS) (Jiang et al., 2024). This prognostic benefit may be attributed to SLC40A1’s regulatory function in modulating immune responses, particularly by enhancing the upregulation of pro-inflammatory mediators such as IL-18 and CCL14, which in turn promote anti-tumor immunity and contribute to delayed disease progression.

At the molecular level, SLC40A1 expression is regulated by both transcriptional and post-transcriptional mechanisms that act in opposition. Post-transcriptionally, miR-147a directly binds to the 3′UTR of SLC40A1 mRNA, leading to translational repression and a subsequent reduction in SLC40A1 protein levels (Xu et al., 2022). In contrast, at the transcriptional level, the cholesterol esterifying enzyme SOAT1 enhances SLC40A1 expression through transcriptional activation, facilitating cellular iron efflux and reducing intracellular iron accumulation (Sun et al., 2023).

Notably, the cell-type-specific expression patterns of SLC40A1 within the GBM tumor microenvironment provide key insights into immune evasion mechanisms associated with the disease. Single-cell RNA sequencing data show that the critical SLC40A1^+^ anti-infection macrophage subpopulation is largely absent in the GBM tumor microenvironment (Hu and Wu, 2021). The loss of this cellular subset may disrupt the balance between local iron homeostasis and immune surveillance, creating a microenvironment that supports tumor cell proliferation and invasion.

#### 2.2.2 Prostate cancer

Prostate cancer remains one of the leading causes of cancer-related mortality in men (DePrimo et al., 2001; Gandaglia et al., 2021; Barry and Simmons, 2017). In studying the molecular mechanisms of prostate cancer, the expression regulation of SLC40A1 exhibits a complex and dynamic pattern characterized by “inhibition–activation–specific association.” These regulatory pathways not only influence iron metabolism in tumor cells but are also closely associated with disease onset, progression, and response to treatment. These mechanisms are supported by both clinical data and experimental models.

From a carcinogenic regulatory perspective, miRNA-mediated inhibition plays a critical role in SLC40A1 dysregulation. Analysis of clinical samples reveals that miR-18a-5p is significantly upregulated in prostate cancer tissues. Mechanistically, miR-18a-5p directly targets the 3′UTR of SLC40A1 mRNA, leading to decreased protein expression. Downregulation of SLC40A1 reduces iron efflux and results in increased intracellular iron accumulation, alleviating iron-dependent growth inhibition and promoting tumor cell proliferation.

*In vitro* experiments further support this mechanism, showing that restoring SLC40A1 expression through gene transfection significantly attenuates the malignant phenotypes of prostate cancer cells, including proliferation and migration. This provides additional evidence for the critical role of the miR-18a-5p-SLC40A1 axis in prostate cancer progression (Liang et al., 2021).

Natural compounds can also modulate SLC40A1 expression and exert anti-cancer effects through specific signaling pathways.

The natural bioactive compound leonurine (Leo) upregulates SLC40A1 expression through transcriptional activation, inducing ferroptosis in tumor cells and effectively suppressing tumor growth in a prostate cancer xenograft model *in vivo*. Leonurine has been demonstrated to induce the upregulation of SLC40A1, and this alteration paradoxically contributes to the promotion of ferroptosis via two distinct yet complementary mechanisms. On one hand, the elevated expression of SLC40A1 uncouples iron efflux from cellular antioxidant defenses, leading to the accumulation of cytosolic labile iron that exceeds the buffering capacity of antioxidant systems such as glutathione. On the other hand, leonurine directly inhibits the activity of glutathione peroxidase 4 (GPX4), thereby impairing the cellular capacity to eliminate lipid peroxides even in the context of enhanced iron export. The cooperative effects of these two processes synergistically amplify ferroptotic signaling cascades.

When SLC40A1 expression was knocked down using siRNA, the anti-cancer effect of Leo was completely abolished, highlighting the essential role of SLC40A1 in Leo’s anti-tumor activity (Liang et al., 2022).

Moreover, SLC40A1 expression is associated with infection, a well-known risk factor for prostate cancer. By integrating bioinformatics analysis with clinical validation, studies have demonstrated that SLC40A1 expression is significantly upregulated in patients with prostate cancer infected with *Neisseria gonorrhoeae* or *Chlamydia trachomatis*. These findings suggest that SLC40A1 may be involved in an interconnected regulatory axis of “infection–ferroptosis–tumorigenesis,” offering a novel perspective on how infectious pathogens may contribute to prostate cancer development through their influence on iron metabolism (Noman et al., 2023).

#### 2.2.3 Clear cell renal cell carcinoma

Clear cell renal cell carcinoma (ccRCC) is the most common histopathological subtype of renal cell carcinoma (Kase et al., 2023; Lam et al., 2004; Kim et al., 2023). In ccRCC, the regulation of SLC40A1 expression and its functional implications are primarily mediated through two molecular mechanisms: miRNA-dependent repression and genetic associations. Both mechanisms are strongly linked to aberrant intratumoral iron metabolism and tumor progression.

miRNA-mediated expression inhibition: miRNA expression profiling has shown that miR-4735-3p is consistently downregulated in ccRCC tissues, with its expression negatively correlating with SLC40A1 levels. *In vitro* transfection experiments demonstrated that miR-4735-3p mimics suppress SLC40A1 protein expression by specifically targeting and binding to the 3′UTR of SLC40A1 mRNA, thereby inhibiting cellular iron efflux (Zhu et al., 2022).

Genetic signature significance: SLC40A1 is part of a fibrosis-associated four-gene signature in ccRCC, including COL1A1, TGF-β1, and VIM. Clinical data analysis indicates that ccRCC patients with elevated expression of this gene signature exhibit increased genomic instability, impaired immune function, and significantly poorer survival outcomes. This strongly suggests a synergistic relationship between SLC40A1-mediated iron export and tumor stromal fibrosis in driving disease progression (Lai et al., 2025).

#### 2.2.4 Pancreatic ductal adenocarcinoma

The mortality rate of pancreatic ductal adenocarcinoma (PDAC) remains high, with limited therapeutic options (Hu et al., 2021; Li et al., 2025; Cabasag et al., 2022). In PDAC, the altered expression and functional roles of SLC40A1 can be evaluated through three interrelated aspects—metabolic regulation, prognosis assessment, and genetic associations—all of which are closely associated with disease initiation and progression.

Metabolic regulation-mediated modulation of gene expression: Sodium β-hydroxybutyrate (Na-OHB), a key metabolite from ketone body metabolism, inhibits the activation of the AMPK-Nrf2 signaling axis by downregulating caveolin-1 (CAV1) expression at the cell membrane. This leads to a reduction in the transcriptional expression of SLC40A1 (Liang et al., 2025).

Prognostic expression characteristics: SLC40A1 is part of a five-gene signature that serves as a robust predictor of recurrence-free survival (RFS) in PDAC patients. Multivariate regression analysis confirmed that elevated SLC40A1 expression is an independent protective factor against postoperative recurrence, with a hazard ratio (HR) of 0.42 (95% confidence interval [CI]: 0.23–0.78), indicating a statistically significant association with prolonged RFS after surgical resection (Feng et al., 2021).

Genetic polymorphism association: Genome-wide association studies (GWAS) combined with pathway analysis have shown that genetic variants in SLC40A1, such as hemojuvelin (HJV), are significantly associated with an increased risk of PDAC (P = 0.002). Carriers of the risk allele have a 1.32-fold higher odds of developing PDAC compared to non-carriers (Julián-Serrano et al., 2021).

#### 2.2.5 Lung cancer (non-small cell lung cancer/lung adenocarcinoma)

Lung adenocarcinoma (LUAD), the most prevalent histological subtype of lung cancer, accounts for approximately 35%–40% of all cases (Wei et al., 2023; Willner et al., 2024; Song et al., 2023). In LUAD, SLC40A1 expression is regulated through three interconnected mechanisms, each closely linked to cellular iron metabolism and disease progression (Gao et al., 2021; Li F. et al., 2021; Miao et al., 2022).

Metabolite-mediated transcriptional inhibition: In LUAD cells, the endogenous metabolite sarcosine activates N-methyl-D-aspartate receptors (NMDAR), leading to the upregulation of the transcriptional repressor MXD3. This represses SLC40A1 transcription, reduces cellular iron export, and results in the accumulation of lipid ROS (Hu et al., 2023).

Natural compound-mediated protein degradation: The plant-derived bioactive compound pomolic acid downregulates SLC40A1 protein expression by enhancing its ubiquitination and subsequent proteasomal degradation. This leads to glutathione (GSH) depletion and Fe^2+^-dependent lipid peroxidation, contributing to tumor progression (Shan et al., 2025).

Cell-specific expression in the tumor microenvironment: Single-cell RNA sequencing, integrated with clinical data, has identified a distinct subpopulation of selenium protein P (SELENOP)^+^ tumor-associated macrophages (TAMs) in LUAD tissues. These TAMs exhibit elevated SLC40A1 expression, which is significantly associated with poor patient prognosis (HR = 1.34, 95% CI: 1.08–1.67). This TAM subset promotes tumor progression by increasing iron availability and secreting immunosuppressive mediators, which facilitate enhanced cancer cell proliferation and immune evasion (Wu et al., 2023).

#### 2.2.6 Hepatocellular carcinoma

Hepatocellular carcinoma (HCC) is one of the most common malignancies globally, representing a significant healthcare challenge (Vogel et al., 2022; Ganesan and Kulik, 2023; Nagaraju et al., 2022). Research into iron metabolism in HCC indicates that SLC40A1 expression exhibits substantial cell-type-specific heterogeneity. This variability is linked not only to disrupted iron homeostasis in tumor cells but also to the regulation of iron metabolism in the peritumoral microenvironment (Shen et al., 2018), offering valuable insights into the mechanisms underlying iron dysregulation in HCC.

Single-cell RNA sequencing analysis of HCC and adjacent non-tumor liver tissues revealed distinct SLC40A1 expression patterns. Compared to normal hepatocytes, HCC cells exhibit significant downregulation of SLC40A1, impairing cellular iron export and leading to intracellular iron accumulation. This accumulation supports tumor proliferation. In contrast, a specific subpopulation of macrophages in peritumoral tissues shows marked upregulation of SLC40A1. These macrophages secrete hepcidin (HAMP), forming an autocrine “HAMP-SLC40A1” regulatory axis that precisely regulates local iron transport and distribution in the liver. This axis helps maintain iron homeostasis in the peritumoral microenvironment and may indirectly suppress tumor progression by inhibiting aberrant hepatocyte proliferation (Hu et al., 2023).

#### 2.2.7 Nasopharyngeal carcinoma

In NPC research, the role of SLC40A1 has evolved from a “clinical diagnostic marker” to a “molecular regulatory target.” The mechanisms and therapeutic potential of its aberrant expression have been increasingly understood through multidimensional studies, especially in relation to Epstein-Barr virus (EBV) infection, epigenetic modifications, and RNA-mediated regulatory networks.

Clinically, SLC40A1 shows significant promise in diagnosing NPC, with expression patterns in both tissue and serum samples providing a solid foundation for disease screening and early detection.

A clinical cohort study comparing NPC patients to a control group with adenoid hypertrophy demonstrated that SLC40A1 mRNA and protein expression levels were significantly reduced in NPC tissues. Furthermore, SLC40A1 expression showed a strong negative correlation with plasma EBV-DNA load in patients (r = −0.62, P < 0.001). This association suggests that SLC40A1 may play a role in EBV-mediated NPC pathogenesis and supports its potential combined use in diagnostic applications. Additional diagnostic performance analysis revealed that the area under the receiver operating characteristic (ROC) curve (AUC) for serum SLC40A1 in diagnosing NPC was 0.902, which increased to 0.913 when combined with EBV-DNA detection, thereby enhancing diagnostic accuracy. These findings suggest that SLC40A1 could serve as an effective molecular tool for early screening and differential diagnosis of NPC.

At the molecular level, the downregulation of SLC40A1 in NPC is primarily driven by two regulatory mechanisms: epigenetic silencing and RNA modification.

At the epigenetic level, hypermethylation of the SLC40A1 gene promoter has been identified as a key factor in its transcriptional silencing. Methylation-specific PCR analysis revealed significantly higher methylation levels in the SLC40A1 promoter region in NPC tissues compared to adjacent non-tumor tissues (P < 0.001). Functional validation experiments demonstrated that treatment of NPC cells with DNA methyltransferase inhibitors, such as 5-azacytidine, reduced promoter methylation, alleviated transcriptional repression, and restored both mRNA and protein expression of SLC4A1 (Zou et al., 2024). Notably, although SLC40A1 transcription and translation are restored, the activity of functional ferroportin is severely compromised by excessive hepcidin expression and subsequent post-translational degradation. This uncoupling creates a molecular bottleneck: the increased iron efflux capacity cannot counteract the accelerated iron influx driven by tumor metabolic reprogramming, ultimately leading to enhanced intracellular iron accumulation. This epigenetic mechanism not only explains the downregulation of SLC40A1 in NPC but also highlights a promising target for epigenetic therapeutic strategies.

At the RNA modification level, the N6-methyladenosine (m^6^A) regulatory network plays a significant role in regulating SLC40A1 expression. The RNA-binding protein IGF2BP2 enhances the stability of ceruloplasmin (CP) mRNA by recognizing m^6^A modification sites on its transcript, which in turn promotes CP protein expression. As CP is involved in iron metabolism, it indirectly suppresses SLC40A1 expression, inhibiting the ferroptosis pathway and favoring NPC cell growth. *In vitro* experiments demonstrated that specific knockdown of IGF2BP2 using siRNA reduced CP mRNA stability, alleviated SLC40A1 suppression, restored ferroptosis, and significantly inhibited NPC cell proliferation (Yuan et al., 2025).

In conclusion, SLC40A1 in NPC functions not only as an EBV-associated diagnostic biomarker but also as a target molecule regulated by epigenetic and RNA modifications. This dual regulatory role opens avenues for advancing both precise diagnostics and targeted therapies for NPC.

#### 2.2.8 Breast cancer

Breast cancer (BCa) remains a major global health challenge affecting women worldwide (Xiong et al., 2025; Dvir et al., 2024; Michaels et al., 2023). Recent studies on the molecular mechanisms of BCa and clinical prognosis evaluation have highlighted the critical role of SLC40A1, with its expression regulation and functional significance now better understood. This gene is not only modulated by miRNAs and various signaling pathways but also plays a pivotal role in ferroptosis-related prognostic stratification.

SLC40A1 in BCa is subject to dual regulation through “post-transcriptional repression and signaling pathway-mediated protein degradation.” At the post-transcriptional level, miR-18a-5p is significantly upregulated in BCa tissues. Mechanistically, it directly binds to the 3′UTR of SLC40A1 mRNA, repressing its translation and reducing SLC40A1 protein expression. Clinical correlation analyses reveal that elevated miR-18a-5p expression is significantly associated with an increased risk of lymph node metastasis and poor prognosis in BCa patients, suggesting that the miR-18a-5p–SLC40A1 axis plays a key role in disease progression (Liang et al., 2021).

At the signaling pathway level, the natural compound β-eudesmol regulates SLC40A1 expression through an alternative mechanism. Specifically, it inhibits the MEK/ERK signaling pathway, which directly influences the stability of the SLC40A1 protein. Inhibition of this pathway enhances the ubiquitination and subsequent degradation of SLC40A1, leading to a reduction in protein levels (Li Z. et al., 2024). This finding introduces a novel molecular target through which natural compounds can regulate iron metabolism in BCa.

The clinical prognosis and functional significance of SLC40A1 are further demonstrated through its role in the ferroptosis-related gene signature, which includes SLC40A1, GPX4, ACSL4, TFRC, and NCOA4. Clinical data show that BCa patients with low expression of this signature exhibit distinct “malignant phenotype clusters.” These include reduced infiltration of immune effector cells (CD8^+^ T cells and NK cells) in the tumor microenvironment, elevated expression of chemotherapy resistance-related genes (such as ABCB1 and ABCG2, which mediate drug efflux), and significantly shorter RFS periods. This confirms that SLC40A1 is a core component of the ferroptosis pathway in BCa and suggests its potential as a key molecular marker linking iron metabolism, the immune microenvironment, and chemotherapy response. These findings offer valuable insights for personalized treatment and prognosis assessment in BCa.

The clinical significance and functional relevance of SLC40A1 are further emphasized by its inclusion in a ferroptosis-related gene signature, specifically a 5-gene signature for BCa comprising SLC40A1, GPX4, ACSL4, TFRC, and NCOA4. Clinical data indicate that BCa patients with low expression of this signature exhibit characteristics of aggressive malignant phenotypes. These include significantly reduced infiltration of immune effector cells, such as CD8^+^ T cells and NK cells, within the tumor microenvironment, increased expression of chemotherapy resistance-related genes (such as ABCB1 and ABCG2, which mediate drug efflux), and markedly shorter RFS periods (Cheng et al., 2023). These findings confirm the central role of SLC40A1 in the ferroptosis pathway in BCa and suggest its potential as a key molecular marker linking dysregulated iron metabolism, immunosuppressive tumor microenvironments, and chemotherapy resistance. This insight supports its utility in personalized treatment strategies and prognostic evaluations in BCa.

#### 2.2.9 Esophageal squamous cell carcinoma

Esophageal cancer ranks as the sixth leading cause of cancer-related mortality globally (Morgan et al., 2022), with esophageal squamous cell carcinoma (ESCC) as the predominant histological subtype (Waters and Reznik, 2022). Recent studies have uncovered the regulatory mechanisms underlying SLC40A1 expression and its clinical implications in ESCC from two critical perspectives: therapeutic targeting and prognostic assessment. Bruceol has been shown to promote the ubiquitination and degradation of the transcription factor Nrf2, thereby reducing its nuclear translocation activity. Since Nrf2 is a key upstream activator of SLC40A1 transcription, its diminished activity leads to suppressed SLC40A1 expression, impairing iron export in ESCC cells (Zhu et al., 2023). This mechanism highlights potential anti-cancer targets of bruceol and provides molecular support for natural drug-based treatments of ESCC.

From a clinical prognosis perspective, SLC40A1’s significance is further supported by its inclusion in a 7-gene immune-related prognostic signature for ESCC, which consists of CD4, CD8A, IFNG, TNF, FOXP3, and IL10. Multivariate Cox regression analysis has confirmed that this signature serves as an independent prognostic factor for ESCC patients (HR = 1.87, 95% CI: 1.32–2.65), effectively distinguishing individuals across different risk categories. Furthermore, analysis of immune cell infiltration revealed a significant positive correlation between SLC40A1 expression levels and CD4^+^ T cell infiltration in ESCC tissues (r = 0.48, P < 0.001) (Liu H. R. et al., 2021). These findings suggest that SLC40A1 may indirectly affect patient prognosis by modulating T cell infiltration within the tumor immune microenvironment, providing a functional rationale for its use as an immune-associated prognostic biomarker in ESCC.

#### 2.2.10 Hematopoietic malignancies

##### 2.2.10.1 T-cell acute lymphoblastic leukemia

T-cell acute lymphoblastic leukemia (T-ALL) is a life-threatening hematologic malignancy, with therapeutic toxicity posing significant challenges to survival outcomes (Foà and Chiaretti, 2022; Davis et al., 2023). Using weighted gene co-expression network analysis (WGCNA) and survival analysis, SLC40A1 was identified as a central hub gene in the ferroptosis pathway in T-ALL. Its downregulation in bone marrow samples from T-ALL patients correlates with poorer OS (HR = 2.13, 95% confidence interval [CI]: 1.28–3.55). Multivariate Cox regression analysis confirmed that SLC40A1 is an independent prognostic factor in T-ALL (Liu H. R. et al., 2021; Tian et al., 2023).

##### 2.2.10.2 Acute myeloid leukemia

In acute myeloid leukemia (AML), the expression of SLC40A1 is closely linked to disease risk stratification, and its regulatory mechanisms offer promising targets for therapeutic strategies (Eshibona et al., 2023). Clinically, a 9-gene prognostic signature based on iron metabolism-related genes, including SLC40A1, TFRC, HFE, HAMP, TFR2, FTH1, FTL, GPX4, and SLC7A11, has been established. Elevated expression of SLC40A1 serves as a key biomarker for high-risk AML status. In patients with intermediate-risk AML, this marker helps refine risk classification and provides key guidance for tailoring individualized treatment approaches in clinical practice (Wysota et al., 2024).

At the molecular level, cobalt-based nanomaterials, like iCoDMSN, activate the NRF2-HMOX1 signaling pathway, downregulating SLC40A1 expression. This process leads to iron accumulation in leukemia cells, making them more sensitive to ferroptosis. This regulatory mechanism opens new avenues for targeted therapeutic strategies in AML.

#### 2.2.11 Solid tumors

##### 2.2.11.1 Ewing sarcoma

In Ewing’s sarcoma (EwS), the expression of specific macrophage subpopulations in the tumor microenvironment has provided new insights into disease progression and clinical outcomes. Notably, the cell-specific expression of SLC40A1 has emerged as a key factor. Integrated single-cell RNA sequencing and immunofluorescence analysis revealed a macrophage subpopulation in the EwS microenvironment co-expressing SLC40A1 and C1QA at elevated levels, with a higher proportion in EwS tissues compared to normal tissues. Survival analysis showed that EwS patients with a greater abundance of this SLC40A1^+^C1QA^+^ macrophage subset had significantly poorer prognosis, with an HR of 1.56 (95% CI: 1.12–2.18). These findings suggest that this macrophage subset may contribute to EwS progression through modulation of iron metabolism or immune regulation in the tumor microenvironment (He et al., 2025).

##### 2.2.11.2 Testicular germ cell tumor

In the study of chemotherapy response in testicular germ cell tumors (TGCT), alterations in SLC40A1 expression have been closely linked to cisplatin resistance, providing valuable insights into intertumoral variations in chemosensitivity and aiding in the development of predictive models. Comparative transcriptomic analysis of cisplatin-sensitive and cisplatin-resistant TGCT cell lines revealed a significant upregulation of SLC40A1 in resistant lines, with a 2.3-fold increase (P < 0.01). Based on its differential expression pattern, SLC40A1 was incorporated into a 14-gene signature developed to predict chemotherapy response in TGCT, emerging as a critical molecular biomarker for evaluating the response of TGCT patients to cisplatin-based chemotherapy (Roška et al., 2020).

##### 2.2.11.3 Tongue squamous carcinoma

Regarding the regulation of resistance to radiotherapy and chemotherapy, the splicing factor PRPF19 binds to the SLC40A1 promoter, driving its transcriptional upregulation. This molecular cascade enhances cellular iron efflux, contributing to increased tolerance to radio-chemotherapy in tongue squamous cell carcinoma cells. *In vitro* experiments demonstrated that knockdown of SLC40A1 using siRNA effectively restored the sensitivity of these cells to both radiotherapy and cisplatin (He et al., 2021).

**FIGURE 3:**
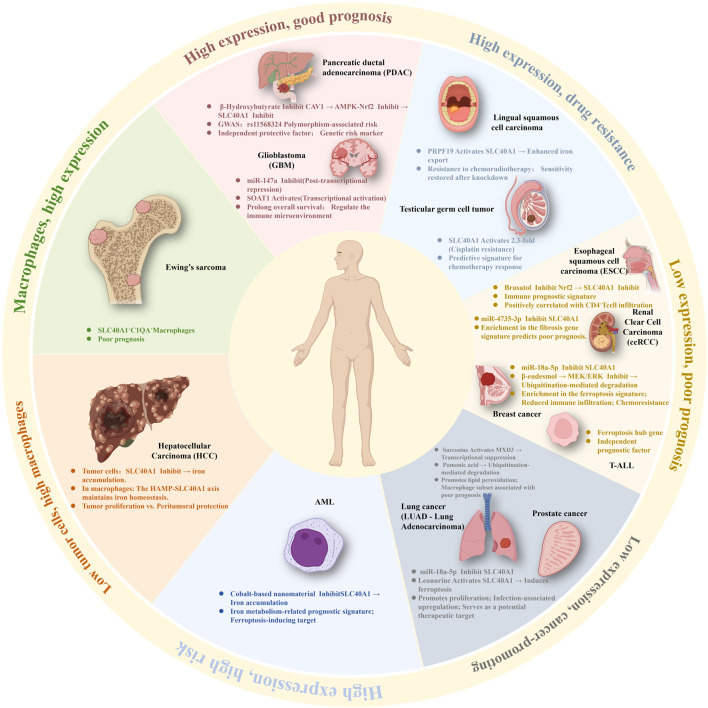
This figure summarizes SLC40A1 expression across 13 tumor types, its regulation, and clinical relevance to prognosis and treatment. SLC40A1 expression varies by cancer type: high levels correlate with better survival in GBM and pancreatic cancer, while low levels are associated with poorer outcomes in many solid tumors, including prostate cancer, renal cancer, lung cancer, and BCa. Regulation occurs at transcriptional, post-transcriptional, epigenetic, and post-translational levels, involving miRNAs, metabolites, natural compounds, signaling pathways, and the tumor microenvironment.

Overall, SLC40A1 exhibits distinct, context-dependent roles across diseases. In non-neoplastic disorders, downregulation or dysfunction consistently drives iron overload, oxidative stress, and ferroptosis-related tissue injury, identifying SLC40A1 as a protective factor. In cancers, by contrast, it displays heterogeneous and even opposing functions shaped by cellular localization, tumor type, and immune microenvironment. It therefore acts neither as a simple oncogene nor a tumor suppressor, but rather as a context-specific prognostic marker, mediator of therapy resistance, and modulator of ferroptosis and tumor progression (Figure 3).

## 3 Clinical translation: therapeutic strategies and diagnostic markers

Given its critical role in both non-tumor disorders, such as iron overload and inflammatory anemia, as well as tumor-related processes, including pan-cancer ferroptosis regulation and immune microenvironment remodeling, SLC40A1 has emerged as a promising therapeutic target for clinical intervention. It also holds substantial potential as a biomarker for disease diagnosis and prognosis assessment (Table 2).

**TABLE 2:** Overview of current SLC40A1-targeted therapeutic approaches, intervention models, and research validation stages in related diseases.

Classification	Disease	Mechanism	Methodology	Drug/intervention	Cell line/animal model	References
Biomarker	Nasopharynx cancer	Serum SLC40A1 suppression, combined with EBV-DNA, achieves a diagnostic AUC of 0.913	Clinical cohort	—	NPC serum	Wang et al. (2025)
	Lung cancer	High SLC40A1 expression and SELENOP-TAM activation link to poor lung cancer prognosis	Single-cell + TCGA	—	Cell line	Wu et al. (2023)
	Breast cancer	The 5-gene ferroptosis signature (low expression of SLC40A1) → inhibition of immune infiltration → drug resistance	Transcriptome	—	Cell line	Cheng et al. (2023)
	Acute T-Cell Lymphoblastic Leukemia (T-ALL)	Low expression of SLC40A1 → Independent poor prognosis	RNA-seq	—	Bone marrow	Zeng et al. (2025)
	Ewing sarcoma	SLC40A1? C1QA? Macrophage activation → Poor prognosis	Single cell	—	Cell line	He et al. (2025)
	Esophageal squamous carcinoma	7-gene immune signature (high expression of SLC40A1) → CD4T cell activation → low risk	qPCR + tissue microarray	—	ESCC	Liu H. et al. (2021)
	Pancreatic cancer	The 5-gene RFS signature (high expression of SLC40A1) → postoperative recurrence inhibition	Bioinformatics + RT-qPCR	—	TCGA	Feng et al. (2021)
Therapy	Clear cell carcinoma of kidney	miR-4735-3p mimic inhibits SLC40A1 → ferroptosis activation → migration inhibition	Cells + animals	miR-4735-3p mimic	Cell line and animal model	Zhu et al. (2022)
	Prostatic cancer	Alkaloid from Leonurus japonicus activates SLC40A1 → ferroptosis is activated → inhibits transplanted tumors	Mouse xenograft model	Leo	Cell line and animal model	Liang et al. (2022)
	Glioblastoma	miR-147a mimic inhibits SLC40A1 → ferroptosis activation → TMZ sensitization	Cells + animals	miR-147a mimic	Cell line and animal model	Xu et al. (2022)
	Pancreatic cancer	Ketone Na-OHB inhibits CAV1 → SLC40A1 inhibition → ferroptosis activation → survival extension	Murine	KPC ketogenic drink	KPC murine	Liang et al. (2025)
	Lung cancer	Pomolic acid inhibits SLC40A1 → GSH inhibition → ferroptosis activation	Cells + animals	Pomoic acid	Cell line and animal model	Ji et al. (2025)
	Esophageal squamous carcinoma	Oleic acid from Brucea javanica inhibits NRF2 → SLC40A1 inhibition → ferroptosis activation	Cells + animals	Baelenol	Cell line and animal model	Zhu et al. (2023)
	Breast cancer	β-eudesmol inhibits MEK/ERK → SLC40A1 inhibition → ferroptosis activation	Cells	β-eudesmol	Human BCa cells	Li Z. et al. (2024)
	Leukemia	Cobalt-based nanoparticles inhibit SLC40A1 → Fe^2+^ activation → ferroptosis activation → radiosensitization	Murine	Cobalt-based nanoparticles	Cell line and animal model	Zhao et al. (2023)
	DN	Acupuncture activates SLC40A1 → inhibits podocyte ferroptosis → inhibits proteinuria	Animals	Acupuncture	Rat model of DKD	Yue et al. (2024)
	ALI/ARDS	Sivelestat activates SLC40A1 → inhibits endothelial ferroptosis → reduces lung injury	Murine	Sivelestat	Mouse model of LPS-ALI	Ren et al. (2024)
	ICH	Cinnamaldehyde activates SLC40A1 → iron efflux is activated → ferroptosis is inhibited → neuroprotection is achieved	Murine	Cinnamyl aldehyde	Mouse model of ICH	Liu Y. et al. (2025)
	Spinal cord injury	Fer-1 activates SLC40A1 → Inhibits neuronal ferroptosis → Motor recovery	Rat	Fer-1	Rat SCIRI	Li S. et al. (2024)
	Anemia of Inflammation	Butyric acid activates SLC40A1 → macrophage iron release activation → anemia relief	Human + murine	Butyrate	Cell line and animal model	Xiao P. et al. (2024)

### 3.1 Therapeutic strategies targeting SLC40A1

Therapeutic strategies targeting SLC40A1 primarily aim to modulate its expression and function to restore iron homeostasis, utilizing approaches such as drug repurposing, epigenetic regulation, and emerging therapies, all with significant translational potential in both non-tumor and tumor contexts.

#### 3.1.1 Drug repurposing (non-oncological and oncological domains)

Drug repurposing, capitalizing on the established safety profiles and shortened R&D timelines of existing drugs, has emerged as a prioritized approach for advancing the clinical application of SLC40A1 modulation. Several approved or extensively studied pharmaceuticals have shown potential to modulate or preserve SLC40A1 expression. In cancer treatment, the primary objective is to enhance tumor cell sensitivity to ferroptosis by regulating SLC40A1, thereby boosting the efficacy of chemotherapy and radiotherapy. For example, acetaminophen has been found to exacerbate iron accumulation in tumor cells by downregulating SLC40A1. Additionally, natural alkaloids from Leonurus japonicus and pomolic acid, a fatty acid derivative, have been shown to interfere with the post-translational modification of SLC40A1, thereby enhancing the cytotoxic effects of ferroptosis inducers such as erastin and RSL3. These findings point to a promising therapeutic strategy for solid tumors, including pancreatic cancer and GBM (Zhao et al., 2023).

#### 3.1.2 Epigenetic and transcriptional regulators

When SLC40A1 expression is aberrantly regulated due to epigenetic silencing (e.g., promoter methylation) or transcriptional repression (e.g., HDAC-mediated regulation), modulators targeting these pathways offer critical therapeutic potential for restoring its functional activity and addressing associated pathological conditions.

HDAC inhibitors: Reversing inflammation-induced transcriptional repression of SLC40A1. Under inflammatory conditions, HDACs, such as HDAC1/3, are recruited to the SLC40A1 promoter, reducing histone acetylation and suppressing SLC40A1 transcription. Pan-HDAC inhibitors, including vorinostat and panobinostat, counteract this repression by restoring histone acetylation and alleviating inflammatory suppression of SLC40A1 expression. In animal models of inflammatory anemia, these inhibitors effectively reduce intracellular iron retention and mitigate anemia-related symptoms, offering a mechanistically grounded therapeutic approach for managing inflammation-related iron metabolism disorders like ACD (Marques et al., 2025).

Nrf2 activators: Regulating SLC40A1 function through transcriptional activation. Nuclear factor erythroid 2-related factor 2 (Nrf2), a key transcription factor that governs cellular antioxidant defense and iron homeostasis, directly binds to the antioxidant response element (ARE) in the SLC40A1 promoter, enhancing its transcription. For instance, bitopertin, a glycine transporter inhibitor originally developed for neuropsychiatric conditions, activates the Nrf2 pathway. By upregulating SLC40A1 expression in hepatic stellate cells, bitopertin promotes intracellular iron export, reduces iron accumulation, and inhibits collagen deposition, significantly alleviating liver tissue injury in animal models of liver fibrosis. These findings suggest bitopertin as a promising candidate for treating metabolic liver diseases, including non-alcoholic steatohepatitis-associated liver fibrosis (Xiao Z. et al., 2024).

DNA demethylating agents: Restoring epigenetically silenced SLC40A1 expression in cancers. In NPC, SLC40A1 is transcriptionally silenced through hypermethylation of its promoter region. DNA methyltransferase inhibitors, such as azacitidine and decitabine, reverse this abnormal methylation, restoring both mRNA and protein expression of SLC40A1. This restoration enhances iron accumulation within tumor cells and increases their sensitivity to ferroptosis, providing a targeted therapeutic approach for tumors with low SLC40A1 expression due to epigenetic abnormalities.

#### 3.1.3 Innovative therapeutic concepts and combination strategies

As the functional mechanisms underlying SLC40A1 are increasingly understood, novel therapeutic strategies targeting its regulatory pathways—such as the induction of ferroptosis and the remodeling of the immune microenvironment—have emerged as key areas of research, offering substantial potential for precise intervention in disease pathogenesis.

Ferroptosis-inducing therapy (for tumor treatment): Inhibiting SLC40A1 function or promoting its degradation increases iron accumulation and induces ferroptosis in tumor cells, making this an important approach in targeted cancer therapy. For instance, strategies like delivering miR-147a mimics (which inhibit SLC40A1 translation) and using SOAT1 inhibitors (which promote SLC40A1 degradation) have been shown in GBM models to enhance chemosensitivity to temozolomide (TMZ). Additionally, the cobalt-based nanomaterial iCoDMSN induces tumor-specific iron overload by activating the NRF2-HMOX1 signaling axis and downregulating SLC40A1 expression, while enhancing the efficacy of radiotherapy. This approach represents a promising combination of nanomedicine with radiotherapy and chemotherapy (Ji et al., 2025).

SLC40A1 stabilizers (for tumor immunotherapy): Maintaining SLC40A1’s iron export function prevents iron overload-induced inactivation of immune effector cells, such as CD8^+^ T cells, while suppressing tumor cell resistance to ferroptosis, thereby fostering an immunologically active “hot tumor” microenvironment. Research is focusing on two main strategies: developing small-molecule inhibitors to block the interaction between hepcidin and SLC40A1 (preventing its internalization and degradation) and designing peptide-based therapeutics to inhibit SQSTM1/p62-mediated autophagic degradation of SLC40A1. These stabilizers have shown synergistic effects with anti-PD-1 immunotherapy in preclinical models, significantly enhancing the therapeutic efficacy of immune checkpoint blockade.

Multi-target combination therapy: Given SLC40A1’s dual role in regulating ferroptosis and immune responses, a promising strategy for clinical intervention may involve “customized triple-combination regimens.” These would include the co-administration of a ferroptosis inducer (e.g., RSL3), an immune checkpoint inhibitor (e.g., anti-PD-1 antibody), and an SLC40A1 modulator (either a stabilizer or inducer), with treatment protocols tailored to the SLC40A1 expression profile and iron metabolic status of different tumor types. For tumors with low SLC40A1 expression, a combination of ferroptosis inducers, immunosuppressive agents, and SLC40A1 inhibitors may be used to amplify ferroptosis. Conversely, for tumors with high SLC40A1 expression but an immunosuppressive microenvironment, a regimen combining a ferroptosis inducer, an immune checkpoint inhibitor, and an SLC40A1 stabilizer could simultaneously enhance antitumor immunity and sensitize cells to ferroptosis.

We will clarify the research validation stage of each SLC40A1-targeted therapeutic strategy in the clinical translation section. We explicitly distinguish three categories of these strategies: those only confirmed in in vitro cell models, those further validated in preclinical *in vivo* animal models (including tumor xenografts and iron-disorder rodent models), and those that remain in the preclinical stage without formal clinical trials. Specifically, the combination of SLC40A1 modulators and temozolomide has currently been verified primarily in GBM cell lines and orthotopic glioma models, with no formal clinical trials conducted to date.

### 3.2 Diagnostic and prognostic biomarkers based on SLC40A1

Genetic variations, epigenetic profiles, and expression levels of SLC40A1 have demonstrated significant potential as specific biomarkers for the early diagnosis, disease classification, and prognosis assessment in a variety of pathological conditions.

#### 3.2.1 Genetic and epigenetic biomarkers for diagnostic detection

Genetic and epigenetic significance of SLC40A1: The genetic and epigenetic characteristics of SLC40A1 position it as a key biomarker for diagnosing rare diseases and advancing research in population genetics. These applications have shown substantial value in clinical diagnostics and epidemiological studies.

Genetic mutation screening: SLC40A1 mutations are considered the gold standard for diagnosing iron transport protein disorders.

Pathogenic mutations in SLC40A1 directly cause HH type 4. Different mutation types lead to distinct functional impairments: loss-of-function mutations (e.g., p.Tyr333His) reduce the iron export activity of SLC40A1, causing intracellular iron accumulation, while gain-of-function mutations (e.g., p.Cys326Ser) result in abnormal enhancement of iron transport. Genetic testing for SLC40A1 mutations is critical for diagnosing and classifying these disorders. Sanger sequencing, which targets specific hotspot mutations, or next-generation sequencing (NGS), which provides comprehensive genetic screening, are both used for precise detection. This precision is essential for informing subsequent clinical decisions, including the development of iron chelation therapy.

Ancestry informative markers (AIMs): A critical tool in population genetics research.

The c.744G>T variant in SLC40A1 displays significant population-specific allele frequency variation, with approximately 5.2% in individuals of West African descent, 5.6% in African Americans, and less than 0.1% in those of European ancestry. This population stratification makes the variant an important ancestral informative marker for inferring West African genetic heritage. Moreover, these frequency differences provide valuable insights into the prevalence of iron metabolism-related disorders, such as hereditary hemochromatosis and iron deficiency anemia, across populations, enhancing the understanding of differential disease susceptibility and supporting the elucidation of the molecular mechanisms behind these variations in prevalence (Barton et al., 2022).

#### 3.2.2 Expression level-based biomarkers

SLC40A1, with its tissue-specific expression and functional relevance in various pathological conditions, has demonstrated substantial potential as a biomarker across multiple clinical applications, including prognosis evaluation, diagnostic refinement, and non-invasive disease monitoring. As such, it provides a critical tool for precision clinical diagnosis and treatment.

Tissue-based prognostic assessment: Low expression of SLC40A1 has consistently been identified as a negative prognostic indicator across numerous malignancies in pan-cancer studies. The quantification of SLC40A1 protein or RNA levels in tumor tissues using immunohistochemistry or RT-PCR reliably predicts key clinical outcomes such as OS and RFS. This relationship has been particularly well-documented in GBM, NPC, and PDAC. In PDAC, a five-gene prognostic model incorporating SLC40A1 surpasses the conventional TNM staging system, offering superior predictive accuracy, better risk stratification, and supporting individualized treatment planning.

Diagnostic refinement: The combination of SLC40A1 with disease-specific biomarkers in a composite panel enhances diagnostic accuracy by overcoming the limitations of single-biomarker approaches. For instance, in NPC, combining SLC40A1 mRNA expression levels with plasma EBV-DNA load results in an AUC of 0.913, significantly outperforming individual biomarkers. This integrated approach provides a highly sensitive and specific method for early detection and differential diagnosis of NPC.

Non-invasive disease monitoring: SLC40A1 shows significant potential in non-invasive diagnostic applications, as demonstrated by clinical studies reporting a detection sensitivity of 90.2%. This biomarker facilitates dynamic monitoring of malignancies such as bladder and esophageal cancer, enabling real-time assessment of treatment efficacy and early detection of disease recurrence. Additionally, it supports the evaluation of inflammatory anemia by overcoming the invasiveness and sampling limitations inherent in traditional tissue biopsy procedures.

Peripheral blood biomarker: SLC40A1 also offers significant advantages as a peripheral blood biomarker. In neurodegenerative disorders like FA, SLC40A1 expression in peripheral blood correlates positively with disease severity. Its measurement is simple and reproducible, making it an ideal peripheral biomarker for neurological disorders, particularly those with central lesions that are challenging to access. This characteristic provides measurable and accessible indicators for monitoring disease progression and evaluating treatment efficacy.

**FIGURE 4:**
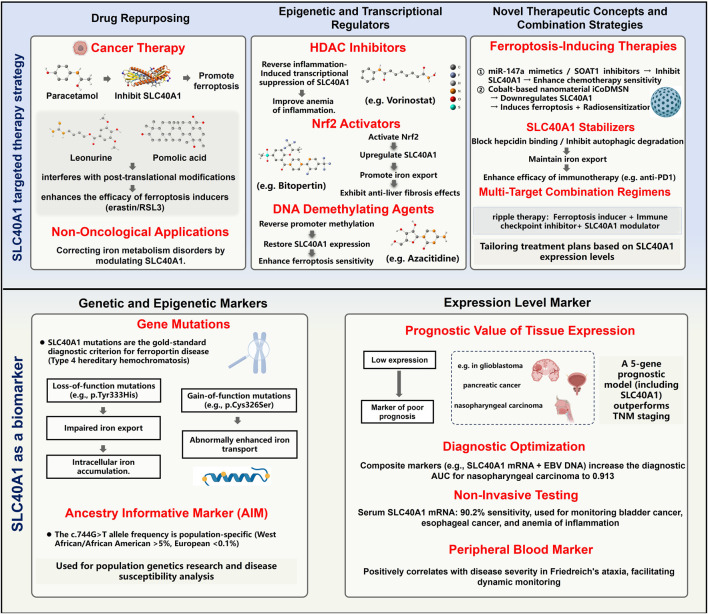
The role of SLC40A1 in diseases: biomarker and therapeutic target. This figure illustrates the key role of SLC40A1 in disease diagnosis and therapy. Top panel: SLC40A1-targeted therapeutic strategies. Bottom panel: SLC40A1 as a biomarker.

In sum, SLC40A1 acts as a context-dependent dual-function regulator. In non-neoplastic diseases, it maintains iron homeostasis; its dysfunction leads to iron overload, inflammation, and tissue damage. In cancers, it modulates ferroptosis, the immune microenvironment, and therapeutic response. It therefore represents a promising therapeutic target and multi-scenario biomarker applicable to genetic diagnosis, pan-cancer risk stratification, and non-invasive liquid biopsy (Figure 4).

## 4 Summary and future outlook

SLC40A1 (FPN), a pivotal regulator of iron metabolism, links systemic iron homeostasis with cellular processes, including ferroptosis and immune responses, highlighting its profound biological importance and potential for clinical application. Its function is highly context-dependent: it maintains iron balance in non-neoplastic tissues, while dysregulation contributes to the development of various disorders such as hereditary hemochromatosis, inflammatory anemia, and neurodegenerative diseases. In the tumor microenvironment, SLC40A1 acts as a tumor suppressor. Downregulation of this protein enhances resistance to ferroptosis and promotes immune evasion, driving tumor progression and resulting in poorer clinical outcomes.

The expression and activity of SLC40A1 are tightly controlled by a multi-layered regulatory network. Genetic mutations directly impair its transport function. At the transcriptional level, key signaling pathways regulate hepcidin expression, while Nrf2 directly activates SLC40A1 transcription. Hepcidin subsequently acts post-translationally to induce ferroportin internalization and degradation. Epigenetic regulation involves dynamic changes in DNA methylation and histone acetylation, whereas post-transcriptional regulation is mediated by miRNAs such as miR-147a that repress SLC40A1 translation. Additionally, post-translational regulation is mediated by an autophagy-dependent degradation pathway involving SQSTM1/p62. Although this complex regulatory network complicates mechanistic studies, it also offers numerous potential targets for clinical intervention.

### 4.1 Summary of the current status of clinical translation

Clinical translation of SLC40A1 has unveiled its broad therapeutic potential. Drug repurposing has recently gained attention, with agents like acetaminophen and melatonin showing promise in alleviating iron overload and oxidative stress in non-neoplastic conditions through the upregulation of SLC40A1. In tumor treatment, a dual-strategy approach is employed: either suppressing SLC40A1 to enhance the efficacy of ferroptosis inducers or upregulating its expression to modulate the tumor immune microenvironment and improve immunotherapy outcomes. In this context, the development of SLC40A1 stabilizers and degradation inhibitors has emerged as a key avenue for next-generation precision-targeted therapeutic strategies. SLC40A1 also serves as a valuable biomarker in medical diagnostics. Genetic mutation analysis of SLC40A1 is the gold standard for diagnosing hereditary hemochromatosis, and its tissue expression levels act as an independent prognostic indicator in cancer. The integration of composite diagnostic panels and liquid biopsy technology has significantly expanded their use in disease diagnosis and monitoring, offering enhanced sensitivity and non-invasive alternatives to traditional methods.

### 4.2 Future research directions and prospects

Despite significant progress, several critical scientific questions and technical challenges remain. Future research should focus on five key areas. First, developing highly specific functional agonists and antagonists for SLC40A1, along with optimizing delivery systems to minimize off-target effects, is crucial. Second, advanced techniques such as cryo-electron microscopy should be applied to analyze the three-dimensional structure of SLC40A1, enhancing the understanding of its structural and functional mechanisms in structural biology. Structural insights into SLC40A1 have been substantially advanced by cryo-electron microscopy (cryo-EM), which has resolved its high-resolution transmembrane topology and the molecular interface for hepcidin binding (Ka et al., 2018). These studies demonstrate that SLC40A1 undergoes a characteristic “open-to-closed” conformational switch upon hepcidin engagement, providing a direct structural explanation for its post-translational regulation. While the present review focuses primarily on disease-related functional mechanisms, further investigations combining cryo-EM structural analysis with functional validation will continue to clarify the allosteric regulation and dynamic conformational changes of ferroportin. Third, the SLC40A1-mediated “iron–immune” axis needs validation across a broader range of cancers and chronic inflammatory diseases. Fourth, the implementation of multicenter, randomized, controlled clinical trials should be accelerated to rigorously evaluate the safety and efficacy of related therapeutic strategies and promote the development of personalized treatments. Fifth, establishing a standardized detection protocol for SLC40A1 mRNA liquid biopsy is essential to integrate this method into routine clinical practice.

In conclusion, SLC40A1 plays a critical role as a central regulatory node in processes such as iron metabolism, cell death regulation, and immune modulation, highlighting its significant potential for advancing disease understanding and facilitating clinical translation. As research progresses and clinical applications expand, targeting SLC40A1 will be increasingly recognized as vital for advancing human health by providing critical therapeutic insights and support.

## References

[B1] Abdalla S. Forghany Z. Ma J. Hollander J. G. Nachane R. Szuhai K. (2025). Identification of novel small molecule inhibitors of ETS transcription factors. FEBS Lett. 599 (12), 1733–1748. 10.1002/1873-3468.70040 40214124 PMC12183613

[B2] Babitt J. L. Lin H. Y. (2011). The molecular pathogenesis of hereditary hemochromatosis. Semin. Liver Dis. 31 (3), 280–292. 10.1055/s-0031-1286059 21901658

[B3] Bao W. D. Pang P. Zhou X. T. Hu F. Xiong W. Chen K. (2021). Loss of ferroportin induces memory impairment by promoting ferroptosis in Alzheimer’s disease. Cell Death Differ. 28 (5), 1548–1562. 10.1038/s41418-020-00685-9 33398092 PMC8166828

[B4] Barry M. J. Simmons L. H. (2017). Prevention of prostate cancer morbidity and mortality: primary prevention and early detection. Med. Clin. North Am. 101 (4), 787–806. 10.1016/j.mcna.2017.03.009 28577627

[B5] Barton J. C. Wiener H. W. Acton R. T. (2022). Estimates of west African ancestry in African Americans using alleles of iron-related genes HJV, SLC40A1, and TFR2. Genet. Test. Mol. Biomarkers 26 (2), 96–102. 10.1089/gtmb.2021.0204 35225679

[B6] Billesbølle C. B. Azumaya C. M. Kretsch R. C. Powers A. S. Gonen S. Schneider S. (2020). Structure of hepcidin-bound ferroportin reveals iron homeostatic mechanisms. Nature 586 (7831), 807–811. 10.1038/s41586-020-2668-z 32814342 PMC7906036

[B7] Britton R. S. Bacon B. R. (2002). Hereditary hemochromatosis and alcohol: a fibrogenic cocktail. Gastroenterol. 122 (2), 563–565. 10.1053/gast.2002.31652 11832468

[B8] Brown N. F. Ottaviani D. Tazare J. Gregson J. Kitchen N. Brandner S. (2022). Survival outcomes and prognostic factors in glioblastoma. Cancers (Basel) 14 (13), 3161. 10.3390/cancers14133161 35804940 PMC9265012

[B9] Cabasag C. J. Ferlay J. Laversanne M. Vignat J. Weber A. Soerjomataram I. (2022). Pancreatic cancer: an increasing global public health concern. Gut 71 (8), 1686–1687. 10.1136/gutjnl-2021-326311 34686577

[B10] Callebaut I. Joubrel R. Pissard S. Kannengiesser C. Gérolami V. Ged C. (2014). Comprehensive functional annotation of 18 missense mutations found in suspected hemochromatosis type 4 patients. Hum. Mol. Genet. 23 (17), 4479–4490. 10.1093/hmg/ddu160 24714983

[B11] Caverzan M. D. Ibarra L. E. (2024). Advancing glioblastoma treatment through iron metabolism: a focus on TfR1 and Ferroptosis innovations. Int. J. Biol. Macromol. 278 (2), 134777. 10.1016/j.ijbiomac.2024.134777 39153669

[B12] Celli B. R. Christenson S. Rabe K. F. Han M. K. van den Berge M. Criner G. J. (2025). Current smoker: a clinical COPD phenotype affecting disease progression and response to therapy. Am. J. Respir. Crit. Care Med. 211 (5), 729–736. 10.1164/rccm.202407-1379CI 39938077 PMC12091029

[B13] Cerveri I. Brusasco V. (2010). Revisited role for mucus hypersecretion in the pathogenesis of COPD. Eur. Respir. Rev. 19 (116), 109–112. 10.1183/09059180.00002710 20956178 PMC9682580

[B14] Chen F. Cai X. Kang R. Liu J. Tang D. (2023). Autophagy-dependent ferroptosis in cancer. Antioxid. Redox Signal. 39 (1-3), 79–101. 10.1089/ars.2022.0202 36734418

[B15] Chen J. Yang X. Li Q. Ma J. Li H. Wang L. (2024). Inhibiting DNA methyltransferase DNMT3B confers protection against ferroptosis in nucleus pulposus and ameliorates intervertebral disc degeneration via upregulating SLC40A1. Free Radic. Biol. Med. 220, 139–153. 10.1016/j.freeradbiomed.2024.05.007 38705495

[B16] Cheng T. C. Wu J. Zhu B. Gao H. Zheng L. Chen W. (2023). Identification of a novel five ferroptosis-related gene signature as a promising prognostic model for breast cancer. J. Cancer Res. Clin. Oncol. 149 (18), 16779–16795. 10.1007/s00432-023-05423-5 37728703 PMC10645672

[B17] Chesseron S. Saidi A. Lecaille F. Lalmanach G. Bigot P. (2023). Alteration of pulmonary epithelial permeability by cathepsin S in chronic obstructive pulmonary disease. Rev. Mal. Respir. 40 (3), 250–253. 10.1016/j.rmr.2023.01.015 36828678

[B18] Davis K. Sheikh T. Aggarwal N. (2023). Emerging molecular subtypes and therapies in acute lymphoblastic leukemia. Semin. Diagn Pathol. 40 (3), 202–215. 10.1053/j.semdp.2023.04.003 37120350

[B19] DePrimo S. E. Shinghal R. Vidanes G. Brooks J. D. (2001). Prevention of prostate cancer. Hematol. Oncol. Clin. North Am. 15 (3), 445–457. 10.1016/s0889-8588(05)70225-2 11525290

[B20] Dlouhy A. C. Bailey D. K. Steimle B. L. Parker H. V. Kosman D. J. (2019). Fluorescence resonance energy transfer links membrane ferroportin, hephaestin but not ferroportin, amyloid precursor protein complex with iron efflux. J. Biol. Chem. 294 (11), 4202–4214. 10.1074/jbc.RA118.005142 30647129 PMC6422103

[B21] Duca L. Granata F. Pierro E. D. Brancaleoni V. Graziadei G. Nava I. (2022). Associated effect of SLC40A1 and TMPRSS6 polymorphisms on iron overload. Metabolites 12 (10), 919. 10.3390/metabo12100919 36295822 PMC9612384

[B22] Dvir K. Giordano S. Leone J. P. (2024). Immunotherapy in breast cancer. Int. J. Mol. Sci. 25 (14), 7517. 10.3390/ijms25147517 39062758 PMC11276856

[B23] Eshibona N. Livesey M. Christoffels A. Bendou H. (2023). Investigation of distinct gene expression profile patterns that can improve the classification of intermediate-risk prognosis in AML patients. Front. Genet. 14, 1131159. 10.3389/fgene.2023.1131159 36865386 PMC9971493

[B24] Feng Z. Chen P. Li K. Lou J. Wu Y. Li T. (2021). A novel ferroptosis-related gene signature predicts recurrence in patients with pancreatic ductal adenocarcinoma. Front. Mol. Biosci. 8, 650264. 10.3389/fmolb.2021.650264 34631790 PMC8495121

[B25] Feng R. Wang D. Li T. Liu X. Peng T. Liu M. (2024). Elevated SLC40A1 impairs cardiac function and exacerbates mitochondrial dysfunction, oxidative stress, and apoptosis in ischemic myocardia. Int. J. Biol. Sci. 20 (2), 414–432. 10.7150/ijbs.89368 38169607 PMC10758104

[B26] Fisher A. L. Phillips S. Wang C. Y. Paulo J. A. Xiao X. Xu Y. (2025). The hepcidin-ferroportin axis modulates liver endothelial cell BMP expression to influence iron homeostasis in mice. Blood 145 (6), 625–634. 10.1182/blood.2024024795 39437541 PMC12782961

[B27] Foà R. Chiaretti S. (2022). Philadelphia chromosome-positive acute lymphoblastic leukemia. N. Engl. J. Med. 386 (25), 2399–2411. 10.1056/NEJMra2113347 35731654

[B28] Galy B. Conrad M. Muckenthaler M. (2024). Mechanisms controlling cellular and systemic iron homeostasis. Nat. Rev. Mol. Cell Biol. 25 (2), 133–155. 10.1038/s41580-023-00648-1 37783783

[B29] Gandaglia G. Leni R. Bray F. Fleshner N. Freedland S. J. Kibel A. (2021). Epidemiology and prevention of prostate cancer. Eur. Urol. Oncol. 4 (6), 877–892. 10.1016/j.euo.2021.09.006 34716119

[B30] Ganesan P. Kulik L. M. (2023). Hepatocellular carcinoma: new developments. Clin. Liver Dis. 27 (1), 85–102. 10.1016/j.cld.2022.08.004 36400469

[B31] Gao X. Tang M. Tian S. Li J. Liu W. (2021). A ferroptosis-related gene signature predicts overall survival in patients with lung adenocarcinoma. Future Oncol. 17 (12), 1533–1544. 10.2217/fon-2020-1113 33432837

[B32] Gu Y. Cao S. Zhu L. Lu G. Tao X. Ding Y. (2025). O-GlcNAc-mediated FTH1 degradation is involved in smoking-induced emphysema through iron ion disorder in alveolar epithelial cells. Ecotoxicol. Environ. Saf. 303, 118999. 10.1016/j.ecoenv.2025.118999 40961600

[B33] Guo P. Li R. Piao T. H. Wang C. L. Wu X. L. Cai H. Y. (2022). Pathological mechanism and targeted drugs of COPD. Int. J. Chron. Obstruct Pulmon Dis. 17, 1565–1575. 10.2147/COPD.S366126 35855746 PMC9288175

[B34] Ha S. Y. Kim J. Choi J. H. (2024). Transcriptional regulation of genetic variants in the SLC40A1 promoter. Korean J. Physiol. Pharmacol. 28 (2), 113–120. 10.4196/kjpp.2024.28.2.113 38414394 PMC10902591

[B35] Hao L. Mi J. Song L. Guo Y. Li Y. Yin Y. (2021). SLC40A1 mediates ferroptosis and cognitive dysfunction in type 1 diabetes. Neuroscience 463, 216–226. 10.1016/j.neuroscience.2021.03.009 33727075

[B36] He F. Ru X. Wen T. (2020). NRF2, a transcription factor for stress response and beyond. Int. J. Mol. Sci. 21 (13). 10.3390/ijms21134777 32640524 PMC7369905

[B37] He Y. Huang C. Cai K. Liu P. Chen X. Xu Y. I. (2021). PRPF19 promotes tongue cancer growth and chemoradiotherapy resistance. Acta Biochim. Biophys. Sin. (Shanghai) 53 (7), 893–902. 10.1093/abbs/gmab059 33954334

[B38] He F. Xu J. Zeng F. Wang B. Yang Y. Xu J. (2025). Integrative analysis of Ewing’s sarcoma reveals that the MIF-CD74 axis is a target for immunotherapy. Cell Commun. Signal 23 (1), 23. 10.1186/s12964-024-02020-y 39800691 PMC11727170

[B39] Helman S. L. Wilkins S. J. McKeating D. R. Perkins A. V. Whibley P. E. Cuffe J. S. M. (2021). The placental ferroxidase zyklopen is not essential for iron transport to the fetus in mice. J. Nutr. 151 (9), 2541–2550. 10.1093/jn/nxab174 34114013

[B40] Honma Y. Karasuyama T. Kumamoto K. Shimajiri S. Toki Y. Tatsumi Y. (2021). Type 4B hereditary hemochromatosis due to heterozygous p.D157A mutation in SLC40A1 complicated with hypopituitarism. Med. Mol. Morphol. 54 (1), 60–67. 10.1007/s00795-020-00259-1 32607777

[B41] Horner R. L. (2023). Targets for obstructive sleep apnea pharmacotherapy: principles, approaches, and emerging strategies. Expert Opin. Ther. Targets 27 (7), 609–626. 10.1080/14728222.2023.2240018 37494064

[B42] Hu L. Wu C. (2021). *In silico* analysis suggests disruption of interactions between HAMP from hepatocytes and SLC40A1 from macrophages in hepatocellular carcinoma. BMC Med. Genomics 14 (1), 128. 10.1186/s12920-021-00977-0 34001107 PMC8130390

[B43] Hu J. X. Zhao C. F. Chen W. B. Liu Q. C. Li Q. W. Lin Y. Y. (2021). Pancreatic cancer: a review of epidemiology, trend, and risk factors. World J. Gastroenterol. 27 (27), 4298–4321. 10.3748/wjg.v27.i27.4298 34366606 PMC8316912

[B44] Hu J. Li Y. Zhang L. Peng G. Zhang F. Zhao X. (2023). Iron overload due to SLC40A1 mutation of type 4 hereditary hemochromatosis. Med. Mol. Morphol. 56 (3), 233–238. 10.1007/s00795-023-00359-8 37382698

[B45] Ji W. Zhang Y. Ji W. Zhang H. Qin B. Xing X. L. (2025). Pomolic acid induces ferroptosis-mediated cell death in non-small cell lung cancer. Front. Pharmacol. 16, 1567942. 10.3389/fphar.2025.1567942 40535769 PMC12174985

[B46] Jiang J. Duan R. Zhu J. Yan J. Ye J. Luo C. (2024). Influence of SLC40A1 on cytokine interactions and immune infiltration in glioblastoma. Neuromolecular Med. 26 (1), 21. 10.1007/s12017-024-08789-y 38750318

[B47] Jończy A. Mazgaj R. Starzyński R. R. Poznański P. Szudzik M. Smuda E. (2020). Relationship between down-regulation of copper-related genes and decreased ferroportin protein level in the duodenum of iron-deficient piglets. Nutrients 13 (1), 104. 10.3390/nu13010104 33396831 PMC7823587

[B48] Ju Z. Jiang Q. Wang J. Wang X. Yang C. Sun Y. (2020). Genome-wide methylation and transcriptome of blood neutrophils reveal the roles of DNA methylation in affecting transcription of protein-coding genes and miRNAs in E. Coli-infected mastitis cows. BMC Genomics 21 (1), 102. 10.1186/s12864-020-6526-z 32000686 PMC6993440

[B49] Ju X. Chen Z. Gao L. Chen M. Wang Q. Jiang Z. (2025). Sputum SLC40A1 as a novel biomarker is increased in patients with acute exacerbation of chronic obstructive pulmonary disease. Int. J. Chron. Obstruct Pulmon Dis. 20, 943–955. 10.2147/COPD.S499176 40191265 PMC11972582

[B50] Julián-Serrano S. Yuan F. Wheeler W. Benyamin B. Machiela M. J. Arslan A. A. (2021). Hepcidin-regulating iron metabolism genes and pancreatic ductal adenocarcinoma: a pathway analysis of genome-wide association studies. Am. J. Clin. Nutr. 114 (4), 1408–1417. 10.1093/ajcn/nqab217 34258619 PMC8488877

[B51] Ka C. Guellec J. Pepermans X. Kannengiesser C. Ged C. Wuyts W. (2018). The SLC40A1 R178Q mutation is a recurrent cause of hemochromatosis and is associated with a novel pathogenic mechanism. Haematologica 103 (11), 1796–1805. 10.3324/haematol.2018.189845 30002125 PMC6278975

[B52] Kane S. F. Roberts C. Paulus R. (2021). Hereditary hemochromatosis: rapid evidence review. Am. Fam. Physician 104 (3), 263–270. 34523883

[B53] Kase A. M. George D. J. Ramalingam S. (2023). Clear cell renal cell carcinoma: from biology to treatment. Cancers (Basel) 15 (3), 665. 10.3390/cancers15030665 36765622 PMC9913203

[B54] Kawabata H. (2018). The mechanisms of systemic iron homeostasis and etiology, diagnosis, and treatment of hereditary hemochromatosis. Int. J. Hematol. 107 (1), 31–43. 10.1007/s12185-017-2365-3 29134618

[B55] Kim H. Park J. S. Choi Z. Min S. Park J Shin S. (2023). Exploring the characteristics of circulating tumor DNA in Pt1a clear cell renal cell carcinoma: a pilot study. Cancers (Basel) 15 (13), 3306. 10.3390/cancers15133306 37444416 PMC10341247

[B56] Koka M. Li H. Akther R. Perlman S. Wong D. Fogel B. L. (2024). Long non-coding RNA TUG1 is downregulated in Friedreich's ataxia. Brain Commun. 6 (3), fcae170. 10.1093/braincomms/fcae170 38846537 PMC11154142

[B57] Lai J. Chen Z. Wu W. (2025). An integrative bioinformatics approach unveils fibrosis-driven prognostic signature and immunotherapeutic potential in kidney renal clear cell carcinoma. Toxicol. Appl. Pharmacol. 504, 117533. 10.1016/j.taap.2025.117533 40850391

[B58] Lam J. S. Belldegrun A. S. Figlin R. A. (2004). Tissue array-based predictions of pathobiology, prognosis, and response to treatment for renal cell carcinoma therapy. Clin. Cancer Res. 10 (18 Pt 2), 6304S–6309S. 10.1158/1078-0432.CCR-sup-040027 15448022

[B59] Li J. Liu J. Xu Y. Wu R. Chen X. Song X. (2021). Tumor heterogeneity in autophagy-dependent ferroptosis. Autophagy 17 (11), 3361–3374. 10.1080/15548627.2021.1872241 33404288 PMC8632302

[B60] Li G. Lin J. Zhang C. Gao H. Lu H. Gao X. (2021). Microbiota metabolite butyrate constrains neutrophil functions and ameliorates mucosal inflammation in inflammatory bowel disease. Gut Microbes 13 (1), 1968257. 10.1080/19490976.2021.1968257 34494943 PMC8437544

[B61] Li F. Ge D. Sun S. L. (2021). A novel ferroptosis-related genes model for prognosis prediction of lung adenocarcinoma. BMC Pulm. Med. 21 (1), 229. 10.1186/s12890-021-01588-2 34256754 PMC8276441

[B62] Li Z. Li J. Liu X. Liu Y. Chen H. Sun X. (2024). β-eudesmol inhibits cell proliferation and induces ferroptosis via regulating MAPK signaling pathway in breast cancer. Toxicon 237, 107529. 10.1016/j.toxicon.2023.107529 38030095

[B63] Liu S. Chen F. Han J. Wang L. Dong Y. (2024). Ferrostatin-1 improves neurological impairment induced by ischemia/reperfusion injury in the spinal cord through ERK1/2/SP1/GPX4. Exp. Neurol. 373, 114659. 10.1016/j.expneurol.2023.114659 38141803

[B64] Li Y. Duan F. Yang S. (2024). SLC40A1-related hemochromatosis associated with a p.Y333H mutation in mainland China: a pedigree report and literature review. BMC Med. Genomics 17 (1), 161. 10.1186/s12920-024-01929-0 38886778 PMC11181628

[B65] Li T. Lin C. Wang W. (2025). Global, regional, and national burden of pancreatic cancer from 1990 to 2021, its attributable risk factors, and projections to 2050: a systematic analysis of the global burden of disease study 2021. BMC Cancer 25 (1), 189. 10.1186/s12885-025-13597-z 39901108 PMC11789343

[B66] Liang B. Zhou C. Cui S. Lu H. Xu R. Xue D. (2021). Upregulation of miR-18a-5p promotes the proliferation of prostate cancer via inhibiting the expression of SLC40A1. Pathol. Res. Pract. 224, 153448. 10.1016/j.prp.2021.153448 34098197

[B67] Liang B. Cui S. Zou S. (2022). Leonurine suppresses prostate cancer growth *in vitro* and *in vivo* by regulating miR-18a-5p/SLC40A1 axis. Chin. J. Physiol. 65 (6), 319–327. 10.4103/0304-4920.365459 36588358

[B68] Liang X. Tian R. Li T. Wang H. Qin Y. Qian M. (2025). Integrative insights into the role of CAV1 in ketogenic diet and ferroptosis in pancreatic cancer. Cell Death Discov. 11 (1), 139. 10.1038/s41420-025-02421-z 40180904 PMC11968908

[B69] Liu L. Li L. Li M. Luo Z. (2021). Autophagy-dependent ferroptosis as a therapeutic target in cancer. ChemMedChem 16 (19), 2942–2950. 10.1002/cmdc.202100334 34110079

[B70] Liu H. R. Jiang G. Z. Xin D. Yang Y. L. Fan Q. X. Meng X. R. (2021). Establishment and validation of prognostic risk score model for esophageal squamous cell carcinoma based on immune related genes. Zhonghua Zhong Liu Za Zhi 43 (6), 666–673. 10.3760/cma.j.cn112152-20200917-00831 34289558

[B71] Liu Y. Yang G. Liu M. Zhang Y. Xu H. Mazhar M. (2025). Cinnamaldehyde and its combination with deferoxamine ameliorate inflammation, ferroptosis and hematoma expansion after intracerebral hemorrhage in mice. J. Neuroinflam. 22 (1), 45. 10.1186/s12974-025-03373-y 39985048 PMC11846400

[B72] Liu S. Tsyplenkova S. Fillebeen C. Pantopoulos K. (2025). Hypoferremic response to chronic inflammation is controlled via the hemojuvelin/hepcidin/ferroportin axis and does not involve hepcidin-independent regulation of Fpn mRNA. Am. J. Hematol. 100 (8), 1323–1333. 10.1002/ajh.27710 40347094 PMC12233014

[B73] Liu Z. Jiao M. Lv J. Han Q. (2025). Increased incidence of chronic obstructive pulmonary disease in women due to long-term passive smoking. Int. J. Chron. Obstruct Pulmon Dis. 20, 2745–2752. 10.2147/COPD.S534060 40791924 PMC12338327

[B74] Lv R. Liu X. Zhang Y. Dong N. Wang X. He Y. (2023). Pathophysiological mechanisms and therapeutic approaches in obstructive sleep apnea syndrome. Signal Transduct. Target Ther. 8 (1), 218. 10.1038/s41392-023-01496-3 37230968 PMC10211313

[B75] Marques O. Horvat N. K. Zechner L. Colucci S. Sparla R. Zimmermann S. (2025). Inflammation-driven NF-κB signaling represses ferroportin transcription in macrophages *via* HDAC1 and HDAC3. Blood 145 (8), 866–880. 10.1182/blood.2023023417 39656097

[B76] McNicholas W. T. Pevernagie D. (2022). Obstructive sleep apnea: transition from pathophysiology to an integrative disease model. J. Sleep. Res. 31 (4), e13616. 10.1111/jsr.13616 35609941 PMC9539471

[B77] Miao H. Ren Q. Li H. Zeng M. Chen D. Xu C. (2022). Comprehensive analysis of the autophagy-dependent ferroptosis-related gene FANCD2 in lung adenocarcinoma. BMC Cancer 22 (1), 225. 10.1186/s12885-022-09314-9 35236309 PMC8889748

[B78] Michaels E. Worthington R. O. Rusiecki J. (2023). Breast cancer: risk assessment, screening, and primary prevention. Med. Clin. North Am. 107 (2), 271–284. 10.1016/j.mcna.2022.10.007 36759097

[B79] Morgan E. Soerjomataram I. Rumgay H. Coleman H. G. Thrift A. P. Vignat J. (2022). The global landscape of esophageal squamous cell carcinoma and esophageal adenocarcinoma incidence and mortality in 2020 and projections to 2040: new estimates from GLOBOCAN 2020. Gastroenterology 163 (3), 649–658.e2. 10.1053/j.gastro.2022.05.054 35671803

[B80] Nagaraju G. P. Dariya B. Kasa P. Peela S. El-Rayes B. F. (2022). Epigenetics in hepatocellular carcinoma. Semin. Cancer Biol. 86 (3), 622–632. 10.1016/j.semcancer.2021.07.017 34324953

[B81] Nemeth E. Ganz T. (2021). Hepcidin-ferroportin interaction controls systemic iron homeostasis. Int. J. Mol. Sci. 22 (12), 6493. 10.3390/ijms22126493 34204327 PMC8235187

[B82] Noman A. A. Islam M. K. Feroz T. Hossain M. M. Shakil M. S. K. (2023). A systems biology approach for investigating significant biomarkers and drug targets common among patients with gonorrhea, chlamydia, and prostate cancer: a pilot study. Bioinform Biol. Insights 17, 11779322231214445. 10.1177/11779322231214445 38033384 PMC10683397

[B83] Peng K. Xiao S. Xia S. Li C. Yu H. Yu Q. (2024). Butyrate inhibits the HDAC8/NF-κB pathway to enhance Slc26a3 expression and improve the intestinal epithelial barrier to relieve colitis. J. Agric. Food Chem. 72 (44), 24400–24416. 10.1021/acs.jafc.4c04456 39440960

[B84] Raha A. A. Biswas A. Henderson J. Chakraborty S. Holland A. Friedland R. P. (2022). Interplay of ferritin accumulation and ferroportin loss in ageing brain: implication for protein aggregation in Down syndrome dementia, Alzheimer’s, and Parkinson’s diseases. Int. J. Mol. Sci. 23 (3), 1060. 10.3390/ijms23031060 35162984 PMC8834792

[B85] Ren J. Deng G. Li R. Jin X. Liu J. Li J. (2024). Possible pharmacological targets and mechanisms of sivelestat in protecting acute lung injury. Comput. Biol. Med. 170, 108080. 10.1016/j.compbiomed.2024.108080 38306776

[B86] Roška J. Wachsmannová L. Hurbanová L. Šestáková Z. Mueller T. Jurkovičová D. (2020). Differential gene expression in cisplatin-resistant and -sensitive testicular germ cell tumor cell lines. Oncotarget 11 (51), 4735–4753. 10.18632/oncotarget.27844 33473258 PMC7771712

[B87] Shan G. Bian Y. Ren S. Hu Z. Pan B. Zeng D. (2025). Sarcosine sensitizes lung adenocarcinoma to chemotherapy by dual activation of ferroptosis via PDK4/PDHA1 signaling and NMDAR-mediated iron export. Exp. Hematol. Oncol. 14 (1), 60. 10.1186/s40164-025-00657-0 40275333 PMC12023509

[B88] Shen Y. Li X. Zhao B. Xue Y. Wang S. Chen X. (2018). Iron metabolism gene expression and prognostic features of hepatocellular carcinoma. J. Cell Biochem. 119 (11), 9178–9204. 10.1002/jcb.27184 30076742

[B89] Sipos D. Raposa B. L. Freihat O. Simon M. Mekis N. Cornacchione P. (2025). Glioblastoma: clinical presentation, multidisciplinary management, and long-term outcomes. Cancers (Basel) 17 (1), 146. 10.3390/cancers17010146 39796773 PMC11719842

[B90] Song Y. Kelava L. Kiss I. (2023). MiRNAs in lung adenocarcinoma: role, diagnosis, prognosis, and therapy. Int. J. Mol. Sci. 24 (17), 13302. 10.3390/ijms241713302 37686110 PMC10487838

[B91] Sun S. Qi G. Chen H. He D. Ma D. Bie Y. (2023). Ferroptosis sensitization in glioma: exploring the regulatory mechanism of SOAT1 and its therapeutic implications. Cell Death Dis. 14 (11), 754. 10.1038/s41419-023-06282-1 37980334 PMC10657441

[B92] Tertre M. L. Ka C. Raud L. Berlivet I. Gourlaouen I. Richard G. (2021). Splicing analysis of SLC40A1 missense variations and contribution to hemochromatosis type 4 phenotypes. Blood Cells Mol. Dis. 87, 102527. 10.1016/j.bcmd.2020.102527 33341511

[B93] Tian C. Zheng M. Lan X. Liu L. Ye Z. Li C. (2023). Silencing LCN2 enhances RSL3-induced ferroptosis in T cell acute lymphoblastic leukemia. Gene 879, 147597. 10.1016/j.gene.2023.147597 37390872

[B94] Tisato V. Castiglione A. Ciorba A. Aimoni C. Silva J. A. Gallo I. (2023). LINE-1 global DNA methylation, iron homeostasis genes, sex and age in sudden sensorineural hearing loss (SSNHL). Hum. Genomics 17 (1), 112. 10.1186/s40246-023-00562-9 38098073 PMC10722762

[B95] Uguen K. Ka C. Collod-Béroud G. Le Tertre M. Guellec J. Férec C. (2023). The spectra of disease-causing mutations in the ferroportin 1 (SLC40A1) encoding gene and related iron overload phenotypes (hemochromatosis type 4 and ferroportin disease). Hum. Mutat. 2023, 5162256. 10.1155/2023/5162256 40225168 PMC11919020

[B96] Uguen K. Tertre M. L. Tchernitchko D. Elbahnsi A. Maestri S. Gourlaouen I. (2024). The dual loss and gain of function of the FPN1 iron exporter results in the ferroportin disease phenotype. HGG Adv. 5 (4), 100335. 10.1016/j.xhgg.2024.100335 39039793 PMC11343060

[B97] Ulasov A. V. Rosenkranz A. A. Georgiev G. P. Sobolev A. S. (2022). Nrf2/Keap1/ARE signaling: towards specific regulation. Life Sci. 291, 120111. 10.1016/j.lfs.2021.120111 34732330 PMC8557391

[B98] Upadhyay P. Wu C. W. Pham A. Zeki A. A. Royer C. M. Kodavanti U. P. (2023). Animal models and mechanisms of tobacco smoke-induced chronic obstructive pulmonary disease (COPD). J. Toxicol. Environ. Health B Crit. Rev. 26 (5), 275–305. 10.1080/10937404.2023.2208886 37183431 PMC10718174

[B99] van Dijck J. Ardon H. Balvers R. K. Bos E. M. Bosscher L. Brouwers H. B. (2025). Survival prediction in glioblastoma: 10-year follow-up from the Dutch neurosurgery quality registry. J. Neurooncol 174 (3), 753–764. 10.1007/s11060-025-05080-3 40410639 PMC12263716

[B100] Vogel A. Meyer T. Sapisochin G. Salem R. Saborowski A. (2022). Hepatocellular carcinoma. Lancet 400 (10360), 1345–1362. 10.1016/S0140-6736(22)01200-4 36084663

[B101] Wang F. Wang J. Shen Y. Li H. Rausch W. D. Huang X. (2022). Iron dyshomeostasis and ferroptosis: a new Alzheimer’s disease hypothesis. Front. Aging Neurosci. 14, 830569. 10.3389/fnagi.2022.830569 35391749 PMC8981915

[B102] Wang H. Qi S. Liu C. Qiao Z. Zhang C. (2025). Differentiating early-stage nasopharyngeal carcinoma from adenoidal hypertrophy via SLC40A1 expression and developing a prognostic model for disease progression. Am. J. Cancer Res. 15 (8), 3434–3448. 10.62347/ZBIH5385 40948531 PMC12432563

[B103] Waters J. K. Reznik S. I. (2022). Update on management of squamous cell esophageal cancer. Curr. Oncol. Rep. 24 (3), 375–385. 10.1007/s11912-021-01153-4 35142974

[B104] Wei T. Zhang M. Zheng X. Xie T. H. Wang W. Zou J. (2022). Interferon-γ induces retinal pigment epithelial cell ferroptosis by a JAK1-2/STAT1/SLC7A11 signaling pathway in Age-related macular degeneration. FEBS J. 289 (7), 1968–1983. 10.1111/febs.16272 34741776

[B105] Wei X. Li X. Hu S. Cheng J. Cai R. (2023). Regulation of ferroptosis in lung adenocarcinoma. Int. J. Mol. Sci. 24 (19), 14614. 10.3390/ijms241914614 37834062 PMC10572737

[B106] Willner J. Narula N. Moreira A. L. (2024). Updates on lung adenocarcinoma: invasive size, grading and STAS. Histopathology. 84 (1), 6–17. 10.1111/his.15077 37872108

[B107] Wu H. Ren X. Ge M. Dong P. Wang S. Yi H. (2022). The novel SLC40A1 (T419I) variant results in a loss-of-function phenotype and may provide insights into the mechanism of large granular lymphocytic leukemia and pure red cell aplasia. Blood Sci. 4 (1), 29–37. 10.1097/BS9.0000000000000099 35399544 PMC8975084

[B108] Wu L. Xia W. Hua Y. Fan K. Lu Y. Wang M. (2023). Cellular crosstalk of macrophages and therapeutic implications in non-small cell lung cancer revealed by integrative inference of single-cell transcriptomics. Front. Pharmacol. 14, 1295442. 10.3389/fphar.2023.1295442 38044943 PMC10690610

[B109] Wysota M. Konopleva M. Mitchell S. (2024). Novel therapeutic targets in acute myeloid leukemia (AML). Curr. Oncol. Rep. 26 (4), 409–420. 10.1007/s11912-024-01503-y 38502417 PMC11021231

[B110] Xiao Z. Kong B. Fang J. Qin T. Dai C. Shuai W. (2021). Ferrostatin-1 alleviates lipopolysaccharide-induced cardiac dysfunction. Bioengineered 12 (2), 9367–9376. 10.1080/21655979.2021.2001913 34787054 PMC8809987

[B111] Xiao P. Cai X. Zhang Z. Guo K. Ke Y. Hu Z. (2024). Butyrate prevents the pathogenic anemia-inflammation circuit by facilitating macrophage iron export. Adv. Sci. (Weinh) 11 (12), e2306571. 10.1002/advs.202306571 38235606 PMC10966513

[B112] Xiao Z. Zhou J. Chen H. Chen X. Wang L. Liu D. (2024). Synthesis, characterization and MAFLD prevention potential of Ganoderma lucidum spore polysaccharide-stabilized selenium nanoparticles. Int. J. Biol. Macromol. 282 (3), 136962. 10.1016/j.ijbiomac.2024.136962 39490485

[B113] Xiong X. Zheng L. W. Ding Y. Chen Y. F. Cai Y. W. Wang L. P. (2025). Breast cancer: pathogenesis and treatments. Signal Transduct. Target Ther. 10 (1), 49. 10.1038/s41392-024-02108-4 39966355 PMC11836418

[B114] Xu P. Ge F. Li W. Xu Z. Wang X. L. Shen J. L. (2022). MicroRNA-147a targets SLC40A1 to induce ferroptosis in human glioblastoma. Anal. Cell Pathol. (Amst) 2022, 2843990. 10.1155/2022/2843990 35942174 PMC9356897

[B115] Xu X. Yu T. Dong L. Glauben R. Wu S. Huang R. (2023). Correction: eosinophils promote pulmonary matrix destruction and emphysema via Cathepsin L. Signal Transduct. Target Ther. 8 (1), 438. 10.1038/s41392-023-01698-9 38012133 PMC10682371

[B116] Yang Q. Liu W. Zhang S. Liu S. (2020). The cardinal roles of ferroportin and its partners in controlling cellular iron in and out. Life Sci. 258, 118135. 10.1016/j.lfs.2020.118135 32712297

[B117] Yeh K. Yeh M. Mims L. Glass J. (2009). Iron feeding induces ferroportin 1 and hephaestin migration and interaction in rat duodenal epithelium. Am. J. Physiol. Gastrointest. Liver Physiol. 296 (1), G55–G65. 10.1152/ajpgi.90298.2008 18974313 PMC3833992

[B118] Yuan Y. Lan Y. Li J. Cui Y. Zhou J. Wen H. (2025). The m6A modification reader protein IGF2BP2 regulates ferroptosis in nasopharyngeal carcinoma cells by stabilizing CP expression via an m6A-dependent mechanism. Biochem. Biophys. Res. Commun. 778, 152417. 10.1016/j.bbrc.2025.152417 40743989

[B119] Yue J. I. Zhang X.-Y. Xiao Y.-M. Zhuang Z.-H. Yang X.-H. Li X.-J. (2024). Acupuncture improve proteinuria in diabetic kidney disease rats by inhibiting ferroptosis and epithelial-mesenchymal transition. Heliyon 10 (13), e33675. 10.1016/j.heliyon.2024.e33675 39071725 PMC11283055

[B120] Zeng X. Liu K. Xu R. Zhang L. Lai P. Du X. (2025). TES and SLC40A1 as potential biomarkers for predicting survival in T-Cell acute lymphoblastic leukemia. Acta Haematol. 148 (2), 163–179. 10.1159/000539435 38806013

[B121] Zhang W. Xu A. Li Y. Zhao S. Zhou D. Wu L. (2019). A novel SLC40A1 p.Y333H mutation with gain of function of ferroportin: a recurrent cause of haemochromatosis in China. Liver Int. 39 (6), 1120–1127. 10.1111/liv.14013 30500107

[B122] Zhang Y. Zou L. Li X. Guo L. Hu B. Ye H. (2024). SLC40A1 in iron metabolism, ferroptosis, and disease: a review. WIREs Mech. Dis. 16 (4), e1644. 10.1002/wsbm.1644 38508867

[B123] Zhao J. Chen Y. Xiong T. Han S. Li C. He Y. (2023). Clustered cobalt nanodots initiate ferroptosis by upregulating heme oxygenase 1 for radiotherapy sensitization. Small 19 (10), e2206415. 10.1002/smll.202206415 36627264

[B124] Zhao S. T. Qiu Z. C. Zeng R. Y. Zou H. X. Qiu R. B. Peng H. Z. (2024). Exploring the molecular biology of ischemic cardiomyopathy based on ferroptosis-related genes. Exp. Ther. Med. 27 (5), 221. 10.3892/etm.2024.12509 38590563 PMC11000445

[B125] Zhou E. H. Zhou T. J. Wang X. T. Zhang J. Y. Guan J. Yin S. K. (2025). Identifying and validating immunological biomarkers in obstructive sleep apnea through bioinformatics analysis. Sci. Rep. 15 (1), 9746. 10.1038/s41598-025-93915-4 40118992 PMC11928569

[B126] Zhu C. Song Z. Chen Z. Lin T. Lin H. Xu Z. (2022). MicroRNA-4735-3p facilitates ferroptosis in clear cell renal cell carcinoma by targeting SLC40A1. Anal. Cell Pathol. (Amst) 2022, 4213401. 10.1155/2022/4213401 35646516 PMC9135554

[B127] Zhu X. Huang N. Ji Y. Sheng X. Huo J. Zhu Y. (2023). Brusatol induces ferroptosis in oesophageal squamous cell carcinoma by repressing GSH synthesis and increasing the labile iron pool via inhibition of the NRF2 pathway. Biomed. Pharmacother. 167, 115567. 10.1016/j.biopha.2023.115567 37742602

[B128] Zou Z. Li R. Huang X. Chen M. Tan J. Wu M. (2024). Identification and validation of immune-related methylated genes as diagnostic and prognostic biomarkers of nasopharyngeal carcinoma. Head. Neck 46 (1), 192–211. 10.1002/hed.27569 37929674

[B129] Zou Z. Wu F. Chen L. Yao H. Wang Z. Chen Y. (2025). The J bs-5YP peptide can alleviate dementia in senile mice by restoring the transcription of Slc40a1 to secrete the excessive iron from brain. J. Adv. Res. 69, 51–59. 10.1016/j.jare.2024.03.014 38527587 PMC11954793

